# FABP4 deactivates NF‐κB‐IL1α pathway by ubiquitinating ATPB in tumor‐associated macrophages and promotes neuroblastoma progression

**DOI:** 10.1002/ctm2.395

**Published:** 2021-05-01

**Authors:** Lei Miao, Zhenjian Zhuo, Jue Tang, Xiaomei Huang, Jiabin Liu, Hai‐Yun Wang, Huimin Xia, Jing He

**Affiliations:** ^1^ Department of Pediatric Surgery, Guangzhou Institute of Pediatrics, Guangdong Provincial Key Laboratory of Research in Structural Birth Defect Disease, Guangzhou Women and Children's Medical Center Guangzhou Medical University Guangzhou China; ^2^ Department of Pathology, Guangzhou Women and Children's Medical Center Guangzhou Medical University Guangzhou China

**Keywords:** FABP4, neuroblastoma, tumor environment, tumor‐associated macrophages

## Abstract

Neuroblastoma (NB) is the most common and deadliest pediatric solid tumor. Targeting and reactivating tumor‐associated macrophages (TAMs) is necessary for reversing immune suppressive state and stimulating immune defense to exert tumoricidal function. However, studies on the function and regulation of TAMs in NB progression are still limited. Fatty acid binding protein 4 (FABP4) in TAMs was correlated with advanced clinical stages and unfavorable histology of NB. FABP4‐mediated macrophages increased migration, invasion, and tumor growth of NB cells. Mechanically, FABP4 could directly bind to ATPB to accelerate ATPB ubiquitination in macrophages. The consequently decreased ATP levels could deactivate NF‐κB/RelA‐IL1α pathway, which subsequently results in macrophages reprogrammed to an anti‐inflammatory phenotype. We also demonstrated that FABP4‐enhanced migration and invasion were significantly suppressed by IL1α blocking antibody. Furthermore, circulating FABP4 was also associated with the clinical stages of NB. Our findings suggest that FABP4‐mediated macrophages may promote proliferation and migration phenotypes in NB cells through deactivating NF‐κB‐IL1α pathway by ubiquitinating ATPB. This study reveals the pathologic and biologic role of FABP4‐mediated macrophages in NB development and exhibits a novel application of targeting FABP4 in macrophages for NB treatment.

## Graphical abstract

1. FABP4 in macrophages expression is correlated with advanced clinical stages and unfavorable histology of neuroblastoma.
2. FABP4 in macrophages increases migration, invasion, and tumor growth of neuroblastoma cells.
3. FABP4 in macrophages may promote proliferation and migration phenotypes in neuroblastoma cells through deactivating NF‐κB‐IL1α pathway by ubiquitinating ATPB.

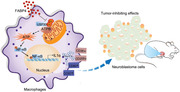

**ABBREVIATIONS**
CMs: conditioned mediums
dt: deletion
ELISA: enzyme‐linked immunosorbent assay
EMT: epithelial‐mesenchymal transition
ER: endoplasmic reticulum
FAO: FA oxidation
FBS: fetal bovine serum
IHC: immunohistochemistry
MMP: matrix metalloproteinases
NB: neuroblastoma
PBMCs: peripheral blood mononuclear cells
TAMs: tumor‐associated macrophages
TCA: tricarboxylic acid
THP1‐DMs: THP1‐derived macrophages
TILs: transition and ineffective lymphocytes
TME: tumor microenvironment
wt: wide type

## 1 INTRODUCTION

Neuroblastoma (NB) is the most common and deadliest pediatric solid tumor.^1^ About 50% of NB patients have metastatic sites (clinical stage 4) such as bone marrow, bone, and liver at diagnosis.^2^ Although the overall prognosis of NB patients has remarkably improved with the advancement in therapeutic strategies such as surgical resection, chemotherapy, and radiotherapy for decades, the event‐free survival rates of high‐risk NB remain less than 50%.^3^ Moreover, distant tumor metastasis, location of tumor near organs, and extensive mesenteric involvement, which are resulted from incomplete therapy, also has a dismal prognosis and lacks effective therapies. However, the tumor‐killing effects of dinutuximab emblematized the success of NB immune therapy and uncovered the role of the immune suppressive tumor microenvironment (TME) as a major obstacle to improve the long‐term survival of children with NB.^4^

Nontumor stromal cells play critical roles in various aspects of tumor progression. Tumor‐associated macrophages (TAMs) are the major stromal component and the most abundant myeloid cells infiltrating to TME,^5^ which exhibit heterogeneous functions for its plasticity.^6^ High density of TAMs is associated with both poor prognosis and decreased survival in multiple cancer types.^7^, ^8^, ^9^ Studies have corroborated that TAMs commonly function as pro‐inflammatory or anti‐inflammatory under TME stimulus where a pro‐ or anti‐tumorigenic environment is created. TAMs are more prevalent in tumors of children with metastatic rather than locoregional NB.^10^ Besides, NB cells also educate TAMs toward immunosuppressive M2‐like phenotype that support their progression and metastatic activity, which in turn worsen the outcome of high‐risk NB tumors.^11^ Blocking macrophage stimulatory factor (CSF‐1) in xenotransplant NB models extends survival in tumor‐bearing mice.^12^ Therefore, targeting TAMs and skewing its function to anti‐tumor effect may become a promising strategy for NB immunotherapy in the future.

The lipid metabolism of tumor cells has been reported as a promising therapeutic target.^13^ In our study, we found that in NB tissues the expressing level of several lipid metabolism‐related genes was higher in the advanced stage than that in the early stage, indicating critical roles of lipid metabolism in NB development. Furthermore, according to our data, fatty acid binding protein 4 (FABP4) expression was more abundant in macrophages compared to other cell types of NB TME. As key mediators involved in metabolism and inflammatory pathways,^14^ FABPs can influence tumor biology through regulating PPAR activity and/or FA uptake and oxidation.^15^ FABP4 is an intracellular lipid chaperone functions to facilitating lipid distribution and response. In addition, FABP4 is also involved in the progression of various cancers. The upregulation of FABP4 is closely associated with enhanced proliferation, migration, and epithelial‐mesenchymal transition (EMT) capacity of cancer cells such as breast, prostate, and ovarian cancer.^16^, ^17^, ^18^ FABP4 also promotes tumor progression by indirectly altering angiogenesis, matrix metalloproteinases (MMP) activity, and cytokine production.^19^ In prostate and ovarian cancers, FABP4 acts as a key mediator between adipocytes and cancer progression.^20^ FABP4 in macrophages is also associated with endoplasmic reticulum (ER) stress and vascular development.^21^, ^22^, ^23^ However, the roles of FABP4 in NB and its effects on NB tumor cells are still unclear.

In this study, we first showed that FABP4 expression was upregulated in NB tissues yet scarcely detected in NB cells. Subsequent data revealed that FABP4 was highly expressed in TAMs and predicted poor survival of patients with NB. We, therefore, hypothesized that only FABP4 that was derived from TAMs instead of NB cells could be a prognostic factor contributing to the pathogenesis of NB. Thus, we aimed to examine the expression profile of FABP4 in TAMs and to further identify the regulatory roles of FABP4‐mediated macrophages during NB progression. This study is the first to report the pathologic and biologic roles of FABP4‐mediated macrophages in NB and suggests that FABP4 in TAMs may be a potential therapeutic target for NB treatment.

## 2 RESULTS

### 2.1 Highly expressed FABP4 in TAMs predicts poor survival of NB patients

**FIGURE 1:**
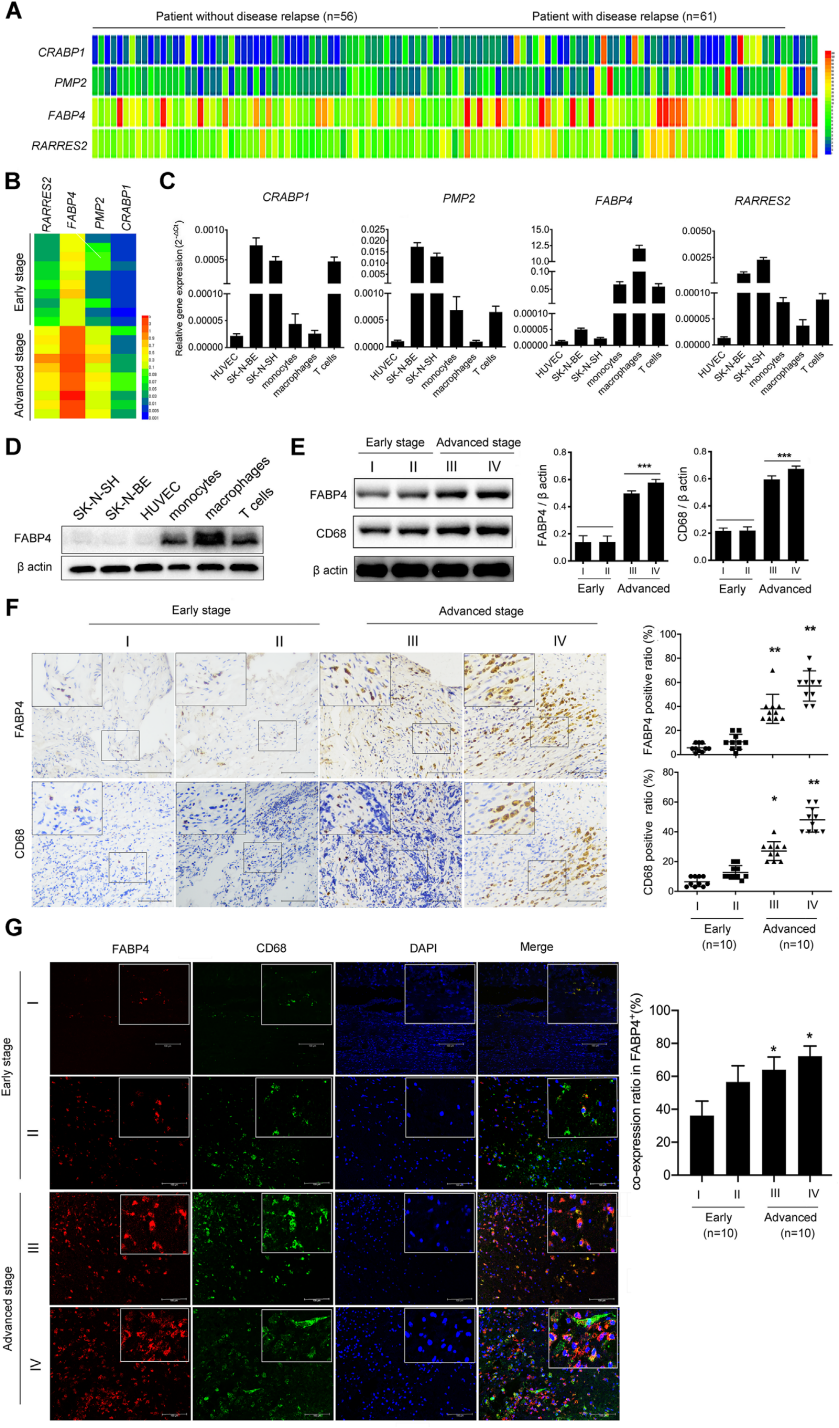
Macrophages with high fatty acid binding protein 4 (FABP4) expression are enriched in the advanced stage of neuroblastoma (NB) patients. (A) The expression levels of genes including *CRABP1*, *PMP2*, *FABP4*, and *RARRES2* in tumors at low‐risk group (patient without disease relapse, *n* = 56) and high‐risk group (patient with disease relapse, including metastases, *n* = 61) of NB in the minimum of 5 years from diagnosis. (B) Heatmap for differentially expressed *CRABP1*, *PMP2*, *FABP4*, and *RARRES2* in 10 pairs of early‐stage (stages I and II) and advanced‐stage (stages III and IV) NB tissue samples. (C) RT‐PCR analysis of *CRABP1*, *PMP2*, *FABP4*, and *RARRES2* in indicated cells mainly existed in the NB environment. (D) Immunoblot analysis showing FABP4 expression in indicated cells. β‐actin was used as a loading control. (E) Left panel, immunoblot analysis showing FABP4 and CD68 expression in early and advanced stage NB tissues. β‐actin was used as a loading control. Right panel, the ratios of FABP4/β‐actin and CD68/β‐actin were quantified, and statistical significance was analyzed. Data are presented as the mean ± SD (*n* = 5). ***p < 0.001. (F) Left panel, representative images of FABP4 and CD68 immunohistochemistry (IHC) staining in different stages of NB tissues. Right panel, the average positive ratio from 3 to 5 fields was counted and identified by symbol and color. *p < 0.05, **p < 0.01. Scale bar represents 100 μm. (G) Representative immunofluorescent staining images and co‐expression level of FABP4 (red) and CD68 (green) in different stages of NB tissues. DAPI (blue) stained for nuclei. *p < 0.05. Scale bar represents 100 μm

To explore the roles of lipid metabolism‐related genes in NB, we first analyzed publicly accessible raw databases from an online microarray dataset (GEO accession number: GSE3446).^24^ Note that 117 NB patients without MYCN gene amplification in this dataset were divided into low‐risk group (patient without disease relapse) and high‐risk group (patient with disease relapse, including metastases) in the minimum of 5 years from diagnosis. We noticed that four genes, *CRABP1*, *PMP2*, *FABP4*, and *RARRES2*, related to the cellular lipid metabolic process, were significantly upregulated in the high‐risk group (Figure 1A and Figure S1A). To confirm whether the four genes identified from tumors without MYCN amplification could also be used as independent factors to predict the outcome of NB children in a clinicopathological way, we then examined the expression of these four genes in 10 NB tissues. The expressions of the four genes were significantly higher in advanced‐stage (stages III and IV) NB tissues than that in early‐stage (stages I and II) NB tissues (Figure 1B). Notably, FABP4 was the most strongly upregulated gene in the advanced stage compared with other genes. Moreover, several genes closely related to lipid metabolism were also upregulated in advanced‐stage NB tissues (Figure S1B), highlighting the importance of lipid metabolism in NB progression.

We wondered whether the four genes that broadly exist in adipocytes^25^ were also functionally expressed in other cell types that existed in the NB environment. Using RT‐PCR, we screened the expression of the above‐altered lipid metabolism‐related genes in NB cell lines (SK‐N‐BE and SK‐N‐SH) as well as NB environment cell lines (HUVEC, monocytes, macrophages, and T cells) and found that FABP4 was enriched in blood‐cell lineages but scarcely expressed in NB cell lines. Of note, FABP4 more highly existed in macrophages than that in CD3^+^ T cells or monocytes (Figure 1C and Figure S1C). The high FABP4 expression level in macrophages was further verified by immunoblotting results (Figure 1D). It is reported that the number of TAMs was closely associated with clinicopathological features of NB.^26^ To confirm the presence of TAMs in NB, we also stained the NB tissues for the macrophage marker CD68. Immunohistochemistry (IHC) and immunofluorescence staining verified that CD68 (macrophage marker) and FABP4 expressions increased gradually from stages I to IV in the NB tissues (Figure 1E–G). Moreover, immunofluorescence staining of NB tissues detected that most of the FABP4 positive staining was localized in CD68^+^ macrophages, and the co‐expression level of FABP4 and CD68 increased gradually (Figure 1G), indicating that the number of infiltrated macrophages might have a positive correlation with FABP4 in NB. The percentages of CD4^+^ T cells in advanced stage NB tissues were significantly higher than that in early‐stage NB tissues. In contrast, the percentages of CD8^+^ T cells, as well as Granzyme B expression, were relatively decreased in advanced‐stage NB tissues (Figure S1D). Taken together, FABP4 was highly expressed in TAMs and predicted poor survival of patients with NB.

### 2.2 FABP4‐mediated macrophages promote NB cells proliferation and migration

**FIGURE 2:**
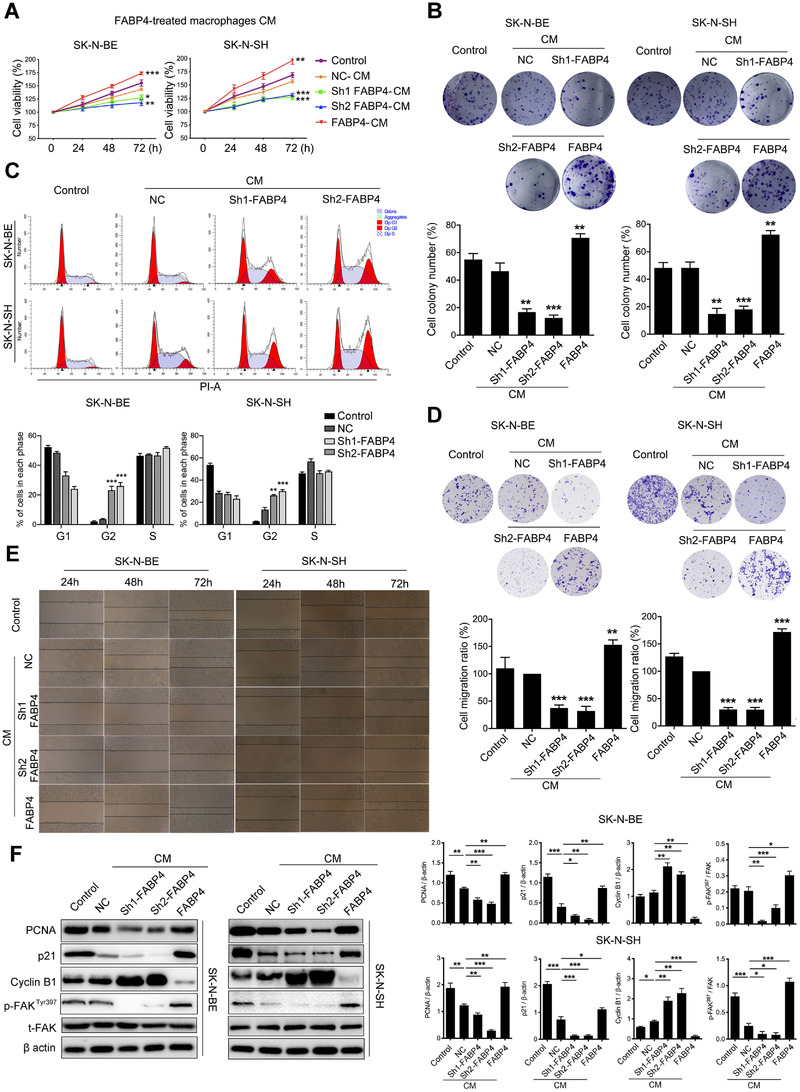
Conditioned Mediums (CMs) of macrophages with knocking‐down fatty acid binding protein 4 (FABP4) have inhibitory effects on the proliferation and migration of neuroblastoma (NB) cells. (A) The proliferation rate of human SK‐N‐BE and SK‐N‐SH cells exposing to CMs of human THP1‐derived macrophages (THP1‐DMs) treated as indicated was assessed by CCK8 assay. (B) Colony formation assay and statistical analysis of human SK‐N‐BE and SK‐N‐SH cells subjecting to CMs of THP1‐DMs treated as indicated. (C) The cell cycle of SK‐N‐BE and SK‐N‐SH cells after subjecting to CMs of FABP4 knocking‐down THP1‐DMs was identified by flow cytometry analysis, and the ratio of cells (%) in each cell cycle phase was statistically analyzed. (D) The transwell assay and statistical analysis of SK‐N‐BE and SK‐N‐SH cells subjecting to CMs of THP1‐DMs treated as indicated. (E) The wound‐healing assay of SK‐N‐BE and SK‐N‐SH cells exposing to CMs of THP1‐DMs treated as indicated for 24, 48, and 72 h, independently. (F) PCNA, p21, Cyclin B1, p‐FAK^397^, and FAK expression levels of SK‐N‐BE and SK‐N‐SH cells were detected and analyzed by Western blot. Data in A‐F are representative of three independent experiments and presented as mean ± SD, *n* = 3 independent samples. *p < 0.05, **p < 0.01, ***p < 0.001. NC stands for the average of undistinguishable controls of scrambled sequence (for Sh‐FABP4) and empty vector (for FABP4 overexpression)

We next determined the effects of macrophages with FABP4 alteration on the progression of NB cells. Human THP1‐derived macrophages (THP1‐DMs) with either knocking‐down or overexpressing FABP4 were established separately. As expected, the expression of FABP4 in THP1‐DMs was reduced or increased correspondingly (Figure S2A). The conditioned mediums (CMs) from various groups of THP1‐DMs were generated and then added to the medium of NB cells. CCK8 and clone formation assays revealed that the proliferation of both SK‐N‐BE and SK‐N‐SH cells were significantly inhibited and promoted, respectively, when cultured with CMs from knocking‐down and overexpression FABP4 macrophages (Figure 2A and B). Considering no observation of significant cell death among these groups, we analyzed the cell cycle progression of NB cells and found that CMs from knocking down FABP4 significantly blocked the G2/M cell cycle transition of SK‐N‐BE and SK‐N‐SH cells (Figure 2C). Meanwhile, migration and wound healing assays revealed that CMs from knocking‐down FABP4 obviously inhibited the migration of SK‐N‐BE and SK‐N‐SH cells, while the FABP4‐overexpressed group showed the opposite effect (Figure 2D and E). We then detected key proteins that are related to cell proliferation (PCNA), G2/M cell cycle arrest (p21, Cyclin B1), and migration (FAK). As expected, CMs of FABP4 knocking‐down inhibited the expressions of PCNA, p21, and phospho‐FAK and increased Cyclin B1 expression in both SK‐N‐BE and SK‐N‐SH cells (Figure 2F). To test whether the effect of FABP4 on NB cells depends on macrophages, SK‐N‐BE and SK‐N‐SH cells with either knocking down or overexpressing FABP4 were also established (Figure S2B). Interestingly, FABP4 knocking down or overexpressing in NB cells did not affect their own cell viability (Figure S2C) and migration ability (Figure S2D). These results suggested that FABP4 might affect NB cell progression depending on macrophages.

### 2.3 FABP4‐mediated macrophages promote NB tumor progression in vivo

To determine whether macrophages‐derived FABP4 represents a general mechanism for promoting tumor progression, we further evaluated tumor growth and metastasis in vivo by using cell line‐derived orthotopic and metastatic NB models, respectively (Figure S3A).

**FIGURE 3:**
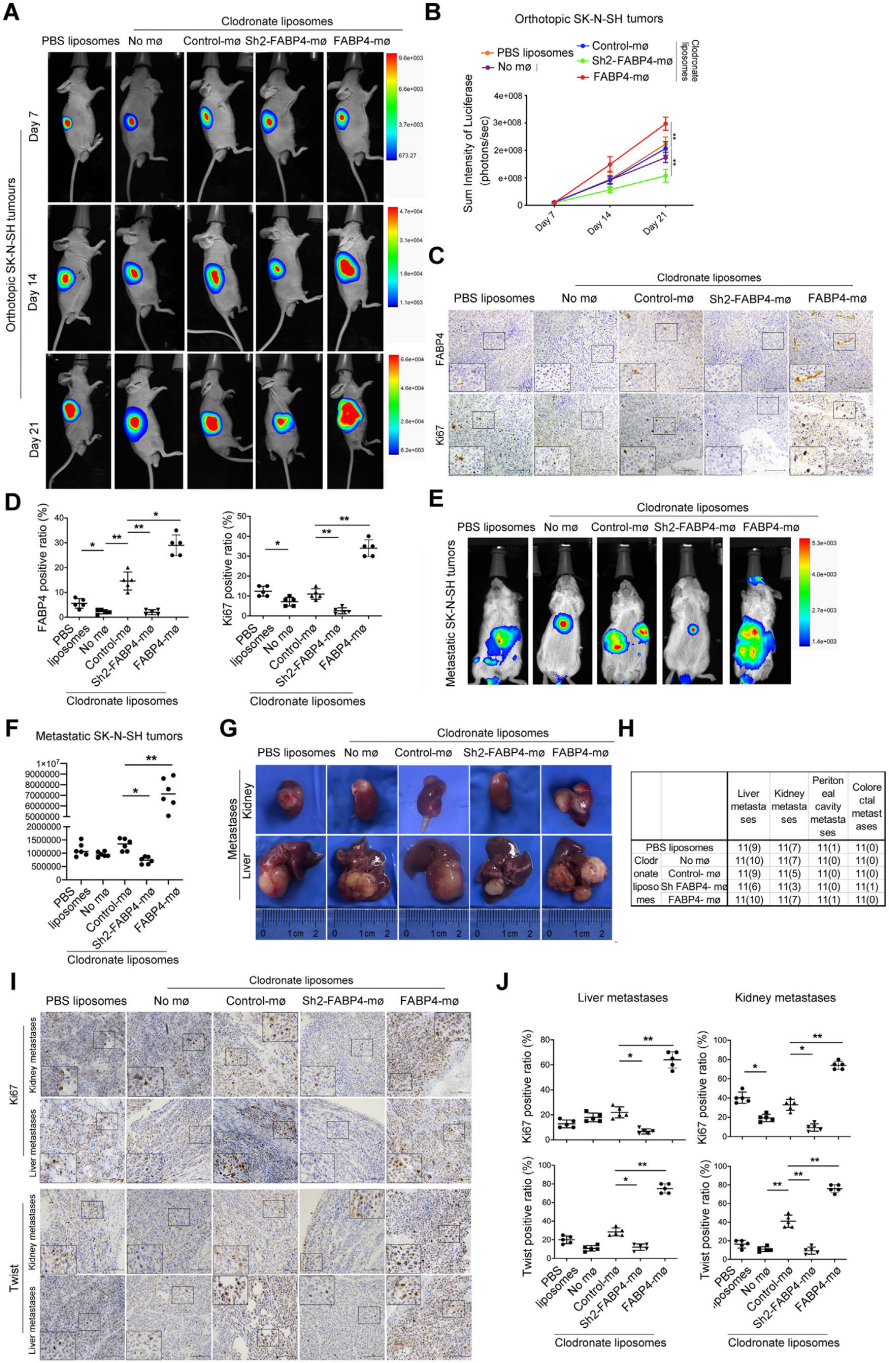
The effects of macrophages with fatty acid binding protein (FABP4) alteration on tumor growth and metastases in vivo. (A) The volumes of SK‐N‐SH‐Luc adrenal orthotopic neuroblastoma (NB). (B) Quantification of light emission was monitored and detected by bioluminescence. The representative images were shown after engrafting peripheral blood mononuclear cells‐derived macrophages (PBMC‐DMs) treated as indicated for days 7, 14, and 21, irrespectively. Data are presented as the mean ± SD (*n* = 6). **p < 0.01. (C) Representative images of Ki67 and FABP4 immunohistochemistry (IHC) staining in NB tissues treated as indicated. Scale bars represent 100 μm. The average positive ratio from 3 to 5 fields was analyzed by symbol, color, and shown. (D) Quantification of (C). *p < 0.05, **p < 0.01. (E) SK‐N‐SH‐Luc cells were injected into NSG mice through the tail vein, and then subjected to PBMC‐DMs treated as indicated. The volumes and sites of metastases were recorded. The representative images were shown after engrafting PBMC‐DMs treated as indicated. (F) Quantification of light emission was detected by bioluminescence. Data are presented as the mean ± SD (*n* = 6). *p < 0.05, **p < 0.01. (G) Visual examination in livers and kidneys metastases of mice treated as indicated. (H) The number of liver, kidney, peritoneal cavity and colorectal metastases treated as indicated were recorded. (I) Representative images of Ki67 and Twist IHC staining in livers and kidneys metastases of NB treated as indicated. Scale bars represent 100 μm. (J) The average positive ratio from 3 to 5 fields was analyzed by symbol, color, and shown. *p < 0.05, **p < 0.01. Control stands for a representative sample of undistinguishable controls of scrambled sequence (for Sh2‐FABP4) and empty vector (for FABP4 overexpression)

The orthotopic model was established by left adrenal gland injection with SK‐N‐SH cells. After irradiation, mice were injected with clodronate liposomes intraperitoneally to deplete macrophages. All the mice were subjected to various groups of CD14^+^ monocytes‐derived macrophages transplantation. The purity of peripheral blood mononuclear cells‐derived macrophages(PBMC‐DMs) was detected by flow cytometry analysis (Figure S3B). First, we observed that endogenous mice macrophages were completely lost in clodronate liposome‐injected mice by F4/80 staining, which were replaced by human macrophages (CD68^+^) after transplantation of PBMC‐DMs (Figure S3C). Besides, the FABP4‐knockdown group has a higher survival rate than the control group (7/10) after clodronate liposome treatment, while the FABP4‐overexpression group has a lower survival rate (6/10) (Figure S3D). Luciferase intensity indicated that tumor growth was repressed by FABP4 knockdown macrophages transplantation, while increased by FABP4 overexpression macrophages transplantation (Figure 3A and B). H&E and IHC staining of NB tissues showed that FABP4 was more expressed in macrophages with FABP4 overexpression group, while seldom observed in the FABP4 knockdown group. Moreover, FABP4 overexpression accelerated in vivo NB tumor growth, as indicated by the upregulation of Ki67 (Figure 3C and D, Figure S3E).

We then took advantage of metastatic NB models by injecting with SK‐N‐SH cells to analyze the influence of FABP4‐altered macrophages on tumor metastasis. Human macrophages were successfully observed in both liver and kidney metastases after transplantation (Figure S3F). Compared to the control group (12/15), we found that the FABP4‐overexpression group has a lower survival rate (11/15), while the FABP4‐knockdown group has no difference (12/15) (Figure S3G). In vivo bioluminescence imaging demonstrated that FABP4 overexpressed macrophages significantly expanded the metastatic tumor size, while FABP4 suppression diminished the metastatic tumor size (Figure 3E and F). Accordingly, a significant increase in the development of liver and kidney node metastases was seen in FABP4 overexpressed macrophages treated mice compared to controls (Figure 3G and H). On the contrary, FABP4 knockdown macrophages significantly decreased liver and kidney node metastases (Figure 3G and H). We also observed that FABP4 overexpression accelerated tumor growth and metastases by IHC staining of Ki67 and TWIST, while FABP4 knockdown exerts the opposite effects (Figure 3I and J, Figure S3H). These data collectively suggest that macrophages‐derived FABP4 plays a critical role in promoting NB progression in vivo.

### 2.4 FABP4‐mediated macrophages promote secretion of IL1α

**FIGURE 4:**
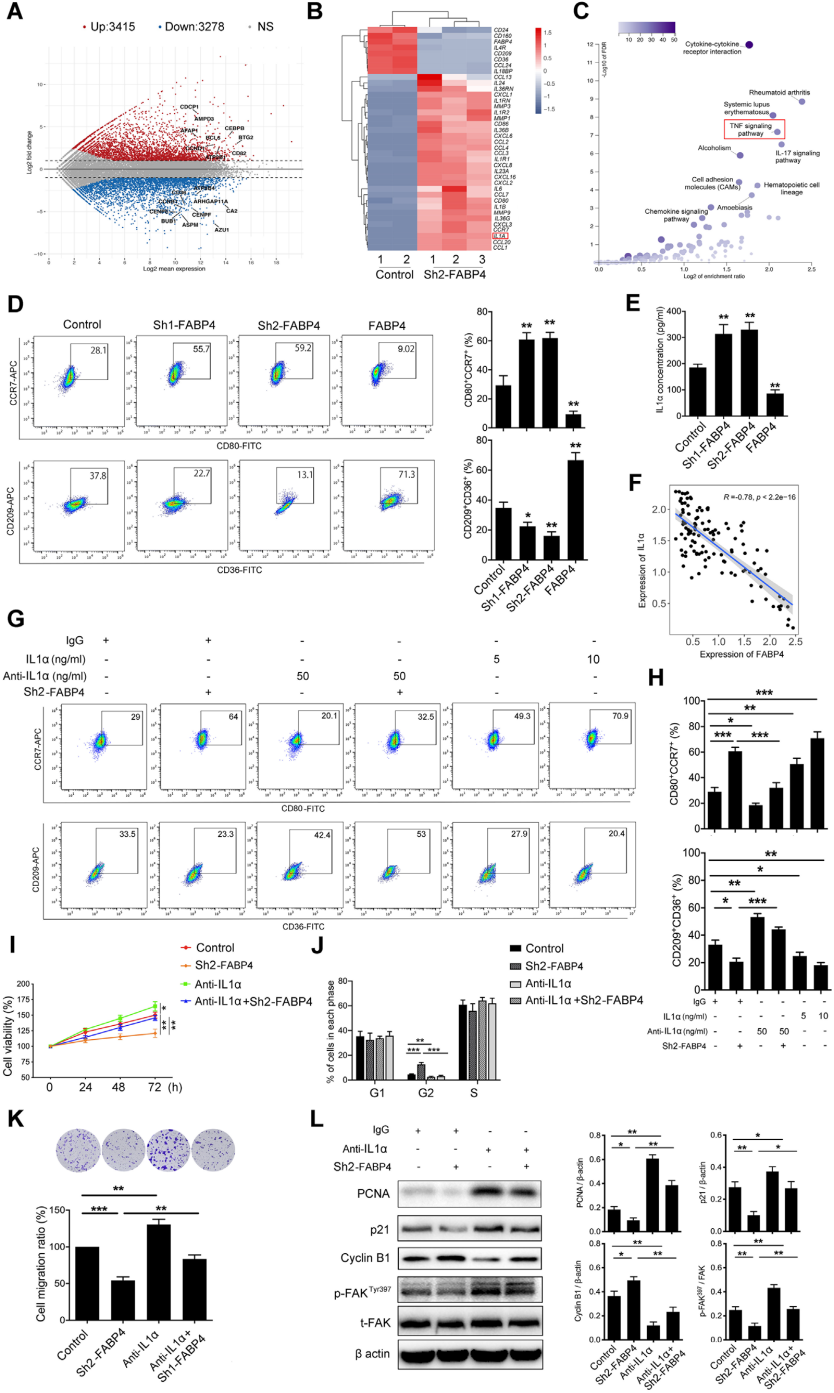
IL1α in conditioned mediums (CMs) from fatty acid binding protein 4 (FABP4) knockdown macrophages increased CD80^+^CCR7^+^ phenotype and is responsible for neuroblastoma (NB) cell proliferation and migration. (A) Volcano plot of RNA‐sequencing expression in FABP4 knockdown‐stimulated human THP1‐derived macrophages (THP1‐DMs). (B) Heatmap of the most upregulated and downregulated inflammation‐related signaling molecules by RNA sequencing expression profiling of THP1‐DMs treated as indicated. (C) KEGG pathway enrichment analysis of RNA sequencing expression profiling in macrophages with FABP4 knockdown. The pathway analysis was performed with the GSEA method, which was based on an empirical permutation test procedure. (D) Left panel, representative flow cytometric plots of CD80^+^CCR7^+^ and CD36^+^CD209^+^ THP1‐DMs treated as indicated. Right panel, quantification of percentage was analyzed. (E) IL1α concentration in the CMs of THP1‐DMs treated as indicated was measured by enzyme‐linked immunosorbent assay (ELISA). (F) Correlation analysis showing expression of FABP4 and IL1α in the online microarray data set (GSE3446). (G) Representative flow cytometric plots of CD80^+^CCR7^+^, CD36^+^CD209^+^ THP1‐DMs treated as indicated. (H) Quantification of percentage was shown and analyzed. (I) The proliferation rate of SK‐N‐SH cells exposing to CMs of THP1‐DMs treated as indicated was assessed by CCK8 assay. (J) The cell cycle of SK‐N‐SH cells after treatment was obtained by flow cytometry analysis, the ratio of cells (%) in each cell cycle phase was statistically analyzed. Representative flow cytometric histograms were showed in Figure S5E. K The transwell assay and statistical analysis of SK‐N‐SH cells subjecting to CMs of human peripheral blood mononuclear cells‐derived macrophages (PBMC‐DMs) treated as indicated. (L) PCNA, p21, Cyclin B1, p‐FAK^397^, and FAK expression levels of SK‐N‐SH cells were detected and analyzed by Western blot. Data in D, E, F, H‐L are presented as the mean ± SD (*n* = 3). *p < 0.05, **p < 0.01, ***p< 0.001. Control stands for the average of undistinguishable controls of scrambled sequence (for Sh‐FABP4) and empty vector (for FABP4 overexpression)

To dig out how FABP4 in macrophages functions on NB cells, we performed RNA sequencing to screen the cytokines altered by FABP4 in THP1‐DMs. A total of 3278 and 3415 genes were downregulated and upregulated, respectively, in NB cells subjected to CMs from FABP4‐knockdown macrophages (Figure 4A and B). To evaluate which signaling pathway mediated the effect of FABP4 in THP1‐DMs, we analyzed the data based on the RNA‐seq using GO and KEGG pathway analyses. The result showed that the TNF signaling pathway was the most enriched signaling pathway (Figure 4C, Figure S4A and B). However, the upregulated genes of macrophages with FABP4 overexpression was significantly enriched in the ECM‐receptor interaction pathway (Figure S4A), which plays a role in cell–substrate junction and has been reported closely related to cancer metastases.^27^

Moreover, RNA sequencing and flow analysis results showed that FABP4 knockdown in macrophages increased the expressions of CD80 and CCR7 (pro‐inflammatory macrophage marker), but decreased the expressions of CD209 and CD36 (anti‐inflammatory macrophage marker), which were all widely expressed on the surface of macrophages. Conversely, FABP4 overexpression exerted the opposite effects (Figure S4C). FABP4‐altered macrophages were further divided into CD80^+^CCR7^+^ and CD209^+^CD36^+^ phenotype according to their surface markers. Knocking down FABP4 significantly increased CD80^+^CCR7^+^ macrophages, but decreased CD209^+^CD36^+^ phenotype. The effects were reversed by FABP4 overexpression (Figure 4D).

We then focused on the cytokines secreted by CMs of FABP4‐knockdown macrophages, which exerted anti‐tumor ability through inhibiting NB cell proliferation and migration. RNA sequencing results revealed multiple cytokines and chemoattractants were upregulated. Considering both CCL1 and CCL20, which screened as the most upregulated chemoattractants, are T cell attracting factors and mainly responsible for Treg cell migration to the TME in human breast and lung cancers,^28^, ^29^, ^30^ we identified other eight differential cytokines (*IL1A, CXCL3, IL1B, IL23A, CXCL1, CCL2, CXCL8*, and *IL6*) with higher local expressions, and verified by RT‐PCR analysis. All of these eight cytokines were upregulated in CMs of FABP4‐knockdown macrophages and downregulated in CMs of FABP4‐overexpression macrophages (Figure S4D). Being the most significantly changed cytokine, interleukin 1α (IL1α), one of the potent inflammatory cytokines that activate the inflammatory process equally to IL1β,^31^ caught our attention. IL1α concentration in CMs of FABP4‐altered macrophages was further detected by enzyme‐linked immunosorbent assay (ELISA). The concentration of IL1α was significantly increased or decreased in CMs after FABP4 knockdown or overexpression in macrophages, respectively (Figure 4E). We also detected a significant reversed correlation (*R* = –0.76) between FABP4 and IL1α expression in the online microarray data set (GEO accession number: GSE3446) (Figure 4F).

### 2.5 FABP4‐mediated macrophages decrease the chemotaxis of CD8^+^ T cells through CXCL8

Considering FABP4 downregulation in TAMs also affects the secretion of several chemokines such as CXCL3, CXCL1, CCL2, and CXCL8 (IL8), we then examined the effects of CMs from knocking‐down or overexpressing FABP4 macrophages on the chemotaxis of T cells. Different CMs from knocking‐down and overexpression FABP4 macrophages were placed at the bottom of the transwell inserts. Purified CD4^+^ or CD8^+^ T cells were seeded in the top chambers. The infiltration of CD8^+^ but not CD4^+^ T cells significantly increased in CMs from FABP4 knocking‐down macrophages when compared to the control, while the chemotactic ability was decreased after exposure to CMs from FABP4 overexpression macrophages (Figure S5A). It has been reported that CXCR1‐expressing CD8^+^ T cells showed high levels of IFN‐γ, perforin, and cytotoxic activity, as well as high responsiveness to IL‐8/CXCL8.^32^ We further performed a function‐blocking assay to demonstrate whether the chemotaxis of CD8^+^ T cell by FABP4 alteration was mediated by CXCL8. As expected, the CXCL8 neutralized antibody inhibited CD8^+^ T‐cell chemoattractant induced by CMs of FABP4 knockdown macrophages. Conversely, the addition of rh CXCL8 dose dependently increased the chemoattractant of CD8^+^ T cells (Figure S5B). Collectively, these results verified that FABP4 deficiency in macrophages not only influenced the property of NB cells but induced CD8^+^ T‐cell chemotaxis through secreting CXCL8 to initiate adaptive immunity of T cells.

### 2.6 FABP4 knockdown regulates macrophage differentiation and tumor‐inhibiting effect through IL1α

We performed a function‐blocking assay to demonstrate the role of IL1α in FABP4‐altered macrophages. The IL1α neutralized antibody inhibited the decrease of CD80^+^CCR7^+^ macrophages and the increase of CD209^+^CD36^+^ macrophages induced by CMs of FABP4 knockdown macrophages. Conversely, the addition of rh IL1α alone showed a dose‐dependently increase of CD80^+^CCR7^+^ macrophages and a decrease of CD209^+^CD36^+^ macrophages (Figure 4G and H), which was further accordingly changed by FABP4 alteration (Figure S6A). Moreover, IL1α depletion in CMs of macrophages enhanced proliferation and migration of SK‐N‐SH cells and blunted the tumor‐inhibiting effects of FABP4‐knockdown macrophages (Figure 4I–K and Figure S6B). Consistently, the expressions of PCNA, p21, phospho‐FAK, and Cyclin B1, which were inhibited or increased by FABP4‐knockdown macrophages, were all rescued by IL1α depletion (Figure 4L). The function of IL1α on NB cells was also investigated. Interestingly, no effect on tumor cell growth or migration in SK‐N‐SH cells after treating IL1α was observed (Figure S6C–E). Collectively, these data demonstrated that FABP4‐induced macrophage polarization, which was mediated by IL1α, is critical for NB progression.

### 2.7 NF‐κB binds to the promoter region of IL1α and regulates its transcriptional activity

**FIGURE 5:**
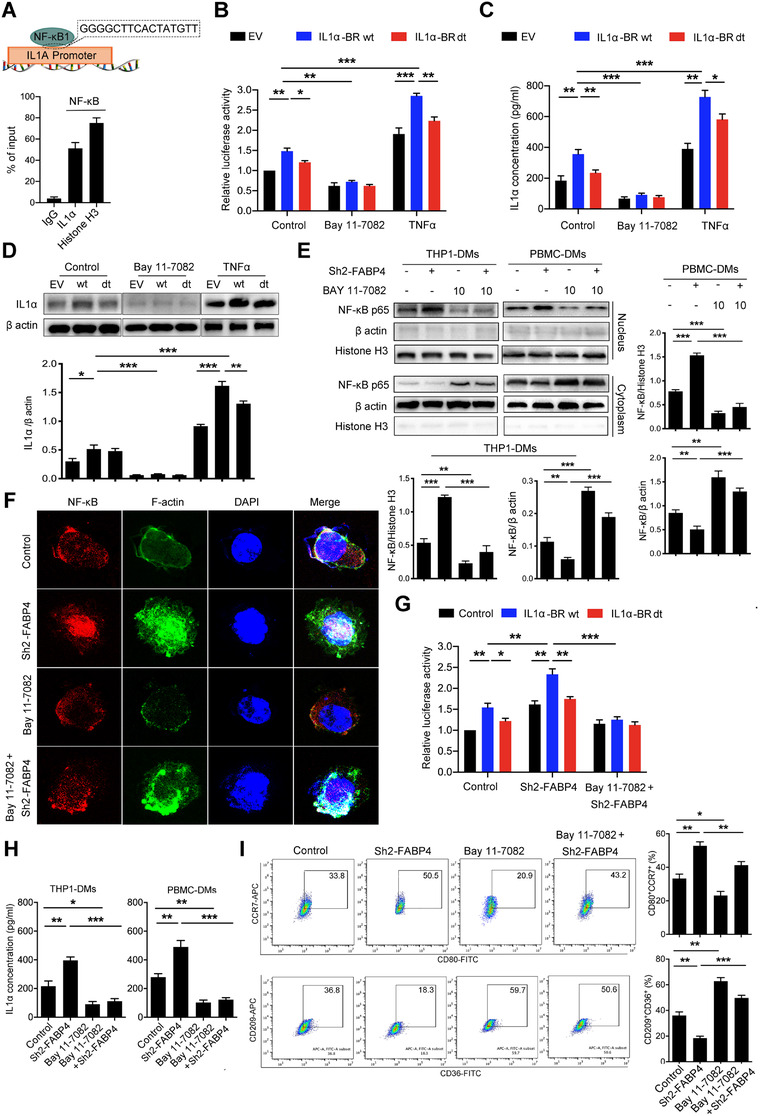
Fatty acid binding protein 4 (FABP4) knockdown‐induced IL1α is transcriptionally regulated by NF‐κB/RelA. (A) Chromatin immuno‐precipitation (ChIP) assays showed that the IL1α promoter contains a consensus NF‐κB binding region. (B) The transcriptional activity of IL1α wide type (wt) and deletion (dt) in human THP1‐derived macrophages (THP1‐DMs) under NF‐κB stimulator or inhibitor treatment was evaluated by luciferase assay. (C) Enzyme‐linked immunosorbent assay (ELISA) for IL1α concentration in THP1‐DMs under NF‐κB stimulator or inhibitor treatment. (D) Upper panel, immunoblot analysis of IL1α expression in THP1‐DMs treated as indicated. β‐actin was used as a loading control. Lower panel, the ratio of IL1α/ β‐actin was quantified. (E) Immunoblot analysis of NF‐κB in the cytoplasm and nuclear of THP1‐ and human peripheral blood mononuclear cells‐derived macrophages (PBMC‐DMs) exposed to Bay 11–7082 treatment. β‐actin and histone H3 were used as loading controls separately in the cell cytoplasm and nuclear. Right panel, the ratios of NF‐κB/ β‐actin and NF‐κB/Histone H3 were quantified, and statistical significance was analyzed. (F) Representative immunofluorescent staining images of NF‐κB (red) and F‐actin (green) in THP1‐DMs exposed to Bay 11–7082 treatment. DAPI (blue) stained for nuclei. Scale bar represents 100 μm. (G) Luciferase activity driven by the IL1α wt and dt promoter in THP1‐DMs treated as indicated. (H) ELISAassay for IL1α concentration in THP1‐DMs treated as indicated. (I) Left panel, representative flow cytometric plots of CD80^+^CCR7^+^ and CD36^+^CD209^+^ PBMC‐DMs treated by Bay 11–7082. Right panel, quantification of percentage was shown and analyzed. Data in A‐E, G‐I are representative of three independent experiments and presented as mean ± SD, *n* = 3 biologically independent samples, *p < 0.05, **p < 0.01, ***p < 0.001

Considering that IL1α was highly expressed in FABP4‐knockdown macrophages, further mechanism was exploited to determine the upstream regulation of IL1α. Numerous sequences of transcript factors involved in the TNF signaling pathway were predicted to bind to the promoter of IL1α by the online Human TFDB database (bioinfo.life.hust.edu.cn/ Human TFDB/).^33^ NF‐κB/RelA, as the downstream regulator of the TNF signaling pathway, was screened with higher binding activity (‐1042–1057 bp) of the IL1α promoter by JASPAR database (version 2020) prediction (Figure S7A).^34^ The binding region of IL1α promoter (IL1α‐BR) to NF‐κB was further confirmed by chromatin immuno‐precipitation (ChIP) assays (Figure 5A). Additionally, we generated overexpressed intact IL‐1α‐BR (IL1α‐BR wt) and IL‐1α‐BR deleted (IL1α‐BR dt) macrophages, then subjected to Bay 11–7082 and TNFα, which functioned as an inhibitor and activator of NF‐κB, respectively (Figure S7B).^35^ According to the luciferase assay, Bay 11–7082 significantly reduced the promoter activity of all the groups, which implies the regulation of NF‐κB to IL1α. Furthermore, the luciferase activity was higher in IL1α‐BR wt than IL1α‐BR dt exposed to Bay 11–7082. However, the luciferase activity was significantly increased after TNFα treatment. The luciferase activity was even higher in macrophages with IL1α‐BR wt, while macrophages with IL1α‐BR dt showed lower activity than IL1α‐BR wt under TNFα (Figure 5B). The alteration of IL1α concentration in the supernatants as well as cellular expression of various groups exerted a similar tendency with luciferase assay results (Figure 5C and D). The results showed that NF‐κB accelerated the transcription activity and expression of IL1α through binding to its promoter region (‐1042–1057 bp).

The role of NF‐κB on tumor‐inhibiting effects of FABP4‐altered macrophages was also investigated. FABP4 knocking down obviously facilitated NF‐κB nuclear translocation and IL1α luciferase activity in macrophages, which were blunted by Bay 11–7082, as demonstrated by Western blot, immunostaining analysis, and luciferase assay (Figure 5E–G). Concurrently, FABP4 overexpression markedly inhibited NF‐κB nuclear translocation, which was rescued by TNFα treatment (Figure S7C and D). Moreover, the increased concentration of IL1α, which was induced by CMs from FABP4 knockdown in macrophages, was also decreased (Figure 5H). Furthermore, Bay 11–7082 reduced CD80^+^CCR7^+^ macrophages while increased CD209^+^CD36^+^ macrophages induced by FABP4 knockdown (Figure 5I). We also observed that macrophages with NF‐κB inhibition promoted the proliferation and migration of SK‐N‐SH cells, and blunted the tumor‐inhibiting effects of FABP4‐knockdown macrophages (Figure S7E–G). These data indicated that macrophages with FABP4‐knockdown promoted CD80^+^CCR7^+^ phenotype and exerted the tumor‐inhibiting effect through NF‐κB/RelA‐mediated IL1α regulation.

### 2.8 ATPB is associated with FABP4‐regulated NF‐κB activation in macrophages

**FIGURE 6:**
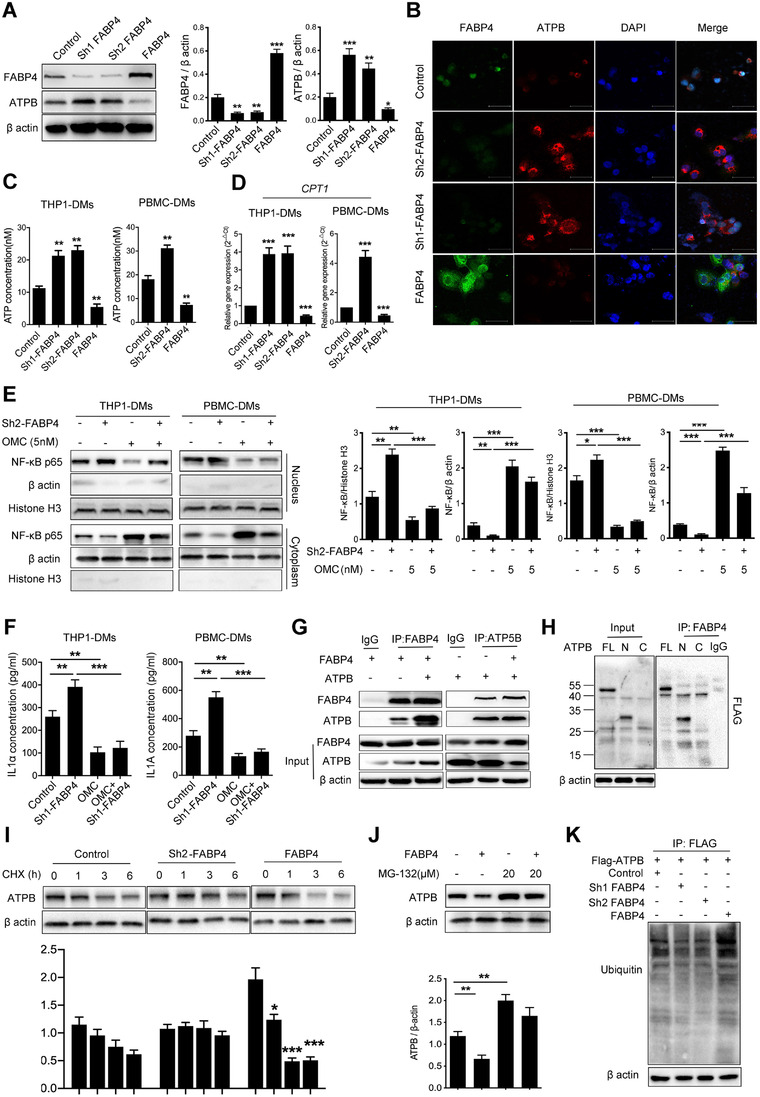
ATPB ubiquitination mediated by fatty acid binding protein 4 (FABP4) is responsible for NF‐κB activation in human THP1‐derived macrophages (THP1‐DMs). (A) Immunoblot analysis of FABP4 and ATPB expression in THP1‐DMs with FABP4 alteration. β‐actin was used as a loading control. Quantification of the immunoblot analysis is shown in the bottom panel. (B) Representative immunofluorescent staining images of ATPB (red) and FABP4 (green) in THP1‐DMs with FABP4 alteration. DAPI (blue) stained for nuclei. Scale bar represents 100 μm. (C) ATP concentration in THP1‐DMs and human peripheral blood mononuclear cells‐derived macrophages (PBMC‐DMs) with FABP4 knockdown and overexpression, respectively. (D) The expression of CPT1 in THP1‐DMs and PBMC‐DMs with FABP4 alteration was detected, respectively. (E) Left panel, immunoblot analysis of NF‐κB expression in cytoplasm and nuclear of THP1‐DMs and PBMC‐DMs treated as indicated. β‐actin and histone H3 were used as loading controls separately in the cell cytoplasm and nuclear. Right panel, the ratios of NF‐κB/ β‐actin and NF‐κB/Histone H3 were quantified, and statistical significance was analyzed. (F) Enzyme‐linked immunosorbent assay (ELISA) for IL1α concentration in THP1‐DMs and PBMC‐DMs with FABP4 knockdown and overexpression. (G) THP1‐DMs were transfected with vectors expressing ATPB and FABP4 and treated with MGC‐132. Co‐immuno‐precipitation (Co‐IP) assays were performed to verify the interaction between ATPB and FABP4. (H) THP1‐DMs were transfected with vectors expressing FLAG‐tagged full‐length (FL), *N*‐terminal (N), or *C*‐terminal (C) constructs. IP and Western blot assays were then performed. (I) Immunoblot analysis detection of ATPB levels in THP1‐DMs with FABP4 alteration, followed by treatment with CHX (50 μg/ml) for the indicated times. Quantification of the immunoblot analysis was shown in the lower panel. (J) THP1‐DMs were treated with MGC‐132 and then subjected to transfection procedures. ATPB expression was detected by immunoblot analysis. Quantification of the immunoblot analysis is shown in the lower panel. (K) FLAG‐tagged ATPB was transfected into THP1 and then subjected to IP assays. The total ubiquitination level after FABP4 alteration was detected by immunoblot analysis. Data in A, C–K are representative of three independent experiments and presented as mean ± SD, *n* = 3 biologically independent samples, *p < 0.05, **p < 0.01, ***p < 0.001. Control stands for the average of undistinguishable controls of scrambled sequence (for shFABP4) and empty vector (for FABP4 overexpression)

To investigate whether FABP4 cooperated with specific proteins to regulate NF‐κB activation, we performed an IP assay, followed by mass spectrometry. According to the MS‐prot score, ATPB ranks as the most potential interactive protein of FABP4 (Figure S8A). As one of the ATP synthases subunits, ATPB is expressed both in the inner mitochondrial membrane and cell surface known as the ectopic expression in normal cells and other cancer cell types and responsible for ATP production.^36^ We first determined the effect of FABP4 on ATPB expression by Western blot and immunostaining analysis. Interestingly, ATPB expression was obviously upregulated by macrophages with FABP4 knockdown, but downregulated by macrophages with FABP4 overexpression (Figure 6A and B). Besides, ATP concentration was altered correspondingly (Figure 6C). We further detected the expression of CPT‐1, a key enzyme initiating mitochondrial fatty acid oxidation. Our results indicated that FABP4 inhibited the expression of CPT‐1 (Figure 6D). Oligomycin A, an ATP synthase inhibitor,^37^ was found to dose‐dependently inhibit ATP production in macrophages (Figure S8B). Further study showed that oligomycin A markedly inhibited the activity of NF‐κB/RelA translocation and the downstream IL1α concentration, which were induced by knocking‐down FABP4 (Figure 6E and F). Our results indicated that ATPB‐mediated ATP production was involved in FABP4 knockdown‐induced NF‐κB activation.

### 2.9 FABP4 binds with the N‐terminal fragment to block the interaction with USP30 and accelerates ATPB ubiquitination

The interaction between FABP4 and ATPB was further confirmed by immunoblotting analyses (Figure 6G). We established three human FLAG‐tagged ATPB vectors harboring FL (1‐529), N‐terminal (N, 1–265), and C‐terminal (C, 266–529) constructs and carried out IP assays using an anti‐FLAG antibody to further identify the ATPB residues that associated with FABP4. Interestingly, we demonstrated that FABP4 interacted mainly with the N‐terminal fragment of ATPB (Figure 6H). The N‐terminal fragment of ATPB contains several protein lysine modification sites, especially ubiquitination (http://plmd.biocuckoo.org/view.php?id = 0003494). FABP4 alteration did not affect the mRNA level of ATPB (Figure S8C). Therefore, we hypothesized whether FABP4 plays a role in regulating ATPB degradation. Cycloheximide was utilized to inhibit the synthesis of proteins. Immunoblotting analyses showed that overexpression of FABP4 markedly accelerated ATPB degradation, which was inhibited by FABP4 knockdown (Figure 6I). In addition, decreased ATPB levels were rescued by the proteasomal inhibitor MG‐132 (Figure 6J) but not by leupeptin (Figure S8D), indicating that the effect of FABP4 on ATPB stabilization was not mainly from lysosome degradation, but proteasomal degradation. Moreover, we performed IP assays in cells overexpressing ATPB and detected ubiquitin levels after FABP4 alteration. Expectedly, the level of ubiquitinated ATPB was significantly decreased by FABP4 knockdown, while increased by FABP4 overexpression in THP1 and NB cells (Figure 6K). E3 ubiquitin ligase March5 directs degradation of import intermediates by proteasomes, and the mitochondrial deubiquitinase (DUB) USP30 are reciprocally associated with the translocase and regulate mitochondrial import. Ubiquitinated intramitochondrial proteins including ATPB lead to the reduction of the TOM (translocase of the outer membrane) complex in USP30‐knockdown cells.^38^ Therefore, we performed co‐IP assays to determine whether FABP4 affects the interaction between ATPB and March5 or USP30. Expectedly, FABP4 upregulation markedly blocked the binding of USP30 to ATPB, while had no effects on the level of March5 (Figure S8E), indicating that FABP4 accelerates ATPB ubiquitination by blocking the interaction of ATPB with USP30. Taken together, our results suggested that FABP4 overexpression increased the binding activity with the N‐terminal fragment of ATPB and impeded the interaction with USP30, leading to ATPB degradation through polyubiquitination.

### 2.10 Levels of FABP4 and IL1α are associated with the phenotype of TAMs and monocytes in NB

**FIGURE 7:**
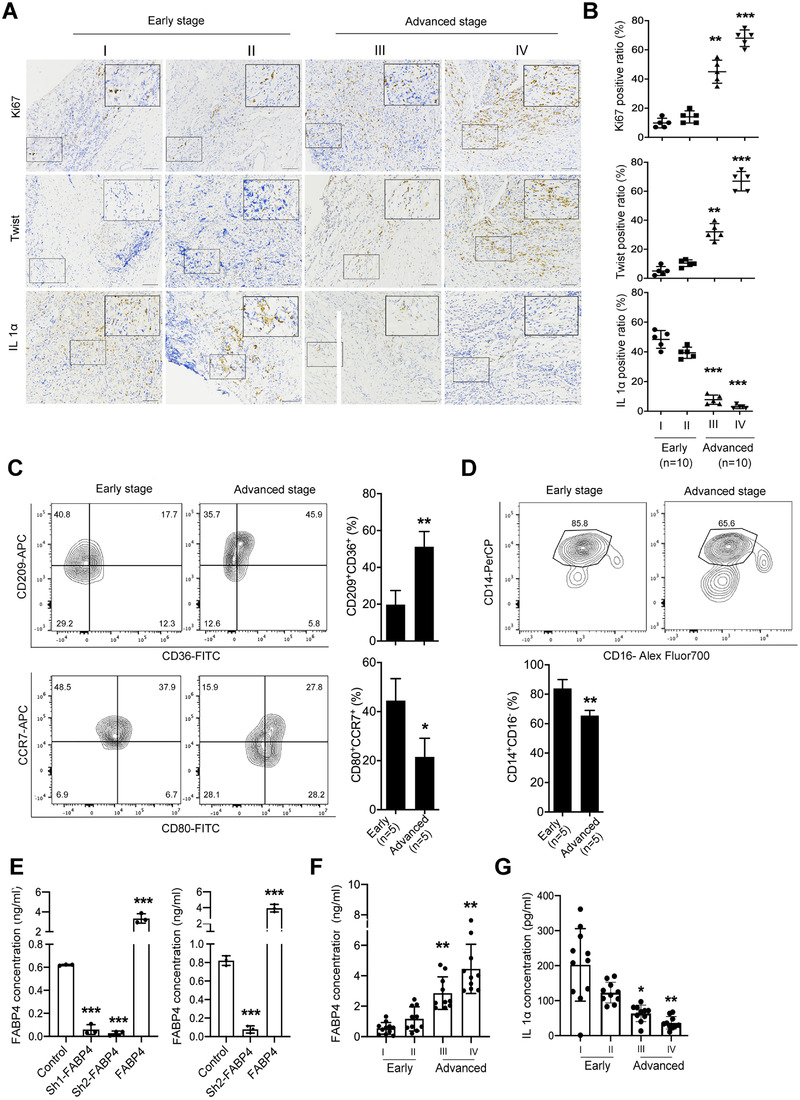
The levels of fatty acid binding protein 4 (FABP4) and IL1α are associated with the phenotype of tumor‐associated macrophages (TAMs) and monocytes in neuroblastoma (NB) tissues and blood from different stages, respectively. (A) Representative images of Ki67, Twist, and IL1α immunohistochemistry (IHC) staining in different stages of NB tissues. The average positive ratio from 3 to 5 fields was counted and identified by symbol and color in (B). **p < 0.01. ***p < 0.001. Scale bar represents 50 μm. (C) Representative flow cytometric plots of CD80^+^CCR7^+^, CD36^+^CD209^+^macrophages in early‐ and advanced‐stage NB tissues. (D) Representative flow cytometric plots of CD14^+^CD16^–^ monocytes in early‐ and advanced‐stage NB tissues. Data in C–D are presented as the mean ± SD (*n* = 5). *p < 0.05, **p < 0.01. (E) Concentration of secreted FABP4 in THP1‐derived macrophages (THP1‐DMs) and human peripheral blood mononuclear cells‐derived macrophages (PBMC‐DMs) with FABP4 knockdown and overexpression, respectively. Data in E are presented as the mean ± SD (*n* = 3). ***p < 0.001. (F) Concentration of secreted FABP4 in early‐stage (stages I and II) and advanced‐stage (stages III and IV) NB samples. (G) Concentration of secreted IL1α in early‐stage (stages I and II) and advanced‐stage (stages III and IV) NB samples. Data in F–G are presented as the mean ± SD (*n* = 10). *p< 0.05, **p < 0.01

We then measured the role of FABP4 in NB plasma (*n* = 40) to correlate the aforementioned results with physiopathology in the clinic. The expression of Ki67 and TWIST was higher in the advanced stage than that of the early stage, reflecting the proliferative and metastatic properties of NB (Figure 7A and B). We also explored the phenotype of tumor‐associated macrophages (TAMs) and classic monocytes in tissues and PBMCs of NB patients, respectively (Figure S9A and B). Our result showed that CD209^+^CD36^+^ macrophages were more existed in advanced stage of NB samples (*n* = 5), compared to the early stage, while CD80^+^CCR7^+^ macrophages were more existed in early stage (Figure 7C). Moreover, CD14^+^CD16^–^ classic monocytes were also higher in PBMCs of early stage, indicating classic monocytes were necessary to TAMs infiltrating and to sustain the inflammatory state in early NB environment (Figure 7D). Considering the concentration of secreted FABP4 in THP1‐DMs and PBMC‐DMs was upregulated and downregulated with FABP4 knockdown and overexpression, respectively (Figure 7E), we then investigated the secreted FABP4 and IL1α in NB patients. As expected, the advanced‐stage group exhibited higher FABP4 levels, whereas lower IL1α levels. On the contrary, the early‐stage group showed the opposite pattern (Figure 7F and G). Moreover, the expression of IL1α was highly expressed in the early stage (Figure 7A and B). Collectively, these data further indicated that FABP4 is a promising prognostic indicator and a potential therapeutic target.

## 3 DISCUSSION

Immune suppressive TME consistently exists during cancer development. It leads to TAMs transition and ineffective lymphocytes (TILs) infiltration and further enlarges immune suppression.^39^ Targeting the TME along with tumor cells has been reported beneficial to a subgroup of high‐risk NB patients.^40^ As one of the major components belonging to the innate immune system, reactivating TAMs is necessary for reversing immune suppressive state and stimulating immune defense to exert tumoricidal function. Our data revealed that CD209^+^CD36^+^ TAMs were more existed in the advanced stage of NB, while CD80^+^CCR7^+^ TAMs as well as CD14^+^CD16^–^ classic monocytes were more existed in tissues and PBMCs of early‐stage NB, indicating the tumor‐inhibiting and tumor‐promoting TME in early and advanced NB environment, respectively. However, the underlying molecular mechanisms responsible for the pro‐tumor activity of TAM in NB remain unclear.

Lipid metabolic process is causally linked to the development of cancers.^41^ However, how the lipid metabolic process is regulated in NB cells and how its role works in the immune alteration of the TME have not been elucidated. Herein, we first obtained the differentially expressed genes targeting lipid metabolism from the online microarray data and further validated the results using clinical NB tissues. FABP4, a lipid metabolism‐related gene, was highly expressed in NB tissues. It is reported that the number of TAMs was strongly associated with an unfavorable histologic type and poor clinical of NB. Further data demonstrated that FABP4 upregulation in NB tissues was primarily restricted to a subset of TAM, but not the NB cell itself. Abundant CD4^+^ T cells infiltration as well as decreased CD8^+^ T cells and granzyme B expression were observed in the advanced stage of NB patients, confirming that the immune‐related status was more passive in the TME of advanced stage and negatively associated with the outcome of NB. Moreover, FABP4 was identified as the most upregulated gene expressed mainly in TAMs of the advanced stage, which is closely associated with inflammatory activation.^21^, ^22^, ^23^, ^42^, ^43^ Circulating FABP4 is also considered as a marker of preclinical metabolic syndrome in humans.^44^ Therefore, we speculated that FABP4 is not only linked to both cellular and systemic metabolic processes but also probably as a clinical or biologic marker associated with the outcome of cancers, especially in NB.

It is known that macrophages with a pro‐inflammatory subtype play a tumor‐inhibiting role during cancer progression.^45^ In our study, FABP4 expression was found to be highly correlated with the expression of CD68 in NB samples. Therefore, high expression of FABP4 in macrophages is associated with poor prognosis in NB and could be used as an independent predictor of NB prognosis. Moreover, we further showed that FABP4 expression in macrophages was critical for proliferation and migration of NB cells in vitro and their growth and metastasis in vivo. Mechanically, lower expression of FABP4 significantly increased CD80^+^CCR7^+^ macrophages (pro‐inflammatory subtype), along with decreased CD209^+^CD36^+^ anti‐inflammatory phenotype, which provides a validated explanation for decreased FABP4 expression in inhibiting NB growth via TAMs. Different chemokine receptors expression leading to various trafficking patterns of T cell subsets, FABP4 deficiency in macrophages also induced CD8^+^ T cell chemotaxis through secreting CXCL8 to initiate adaptive immunity of T cells, which bridges the gap between the innate and acquired immune responses in TME of NB. Thus, our data suggest that FABP4 may serve as a new functional marker for protumor macrophages.

The precise mechanism of FABP4 regulation in macrophages was further investigated. We noticed that FABP4 knockdown stimulated secretion of series of cytokines that are probably responsible for the proinflammatory TME. Although TNF signaling pathway was significant enriched by KEGG analysis, TNF‐α‐stimulated cytokines were not altered according to RNA‐seq results. IL1α was significantly upregulated by FABP4 knocking down. IL1 family and TNF are both potent activators of NF‐κB to induce inflammation. IL1α was screened as the mediator for its potent inflammatory activity and significant negative correlation with FABP4 in the GEO database. We thus hypothesized that IL1α, but not TNF‐α, may be responsible for FABP4‐induced function, and verified through further studies. The roles of IL1α in cancer biology are various, with either a pro‐tumorigenic role or tumor suppressor.^46^ Overexpression of IL1α in fibrosarcoma lines appears to induce antitumor immunity mediated by innate immune cells.^47^ Furthermore, IL1α blocking antibody significantly suppresses macrophages specific FABP4‐enhanced NB cell migration and invasion. In our study, we demonstrated that IL1α stimulated by FABP4 knockdown takes the main responsibility of macrophages phenotype skewing and anti‐tumor ability.

Given that the TNF signaling pathway is significantly enriched in macrophages with FABP4 knockdown, transcriptional candidates have a binding activity of the IL1α promoter. IL1α‐BR were predicted and screened through associating with the TNF pathway. Expectedly, the transcriptional factor NF‐κB/RelA shows highly binding activity of the IL1α promoter (IL1α‐BR) and increases IL1α expression. These data revealed the inflammatory role of the NF‐κB/RelA‐IL1α axis in FABP4 regulation. Numerous proteins have been reported to interact with FABP4, such as HSL and cytokeratin 1.^48^ We proposed that FABP4 may regulate the transcriptional activity of NF‐κB/RelA through protein interaction. Interestingly, mass spectrometry analysis and Co‐IP study identified ATPB as a newly discovered FABP4‐binding protein. ATPB, known as a subunit of mitochondrial ATP synthase, is responsible for catalyzing ATP synthesis and promoting oxidative phosphorylation.^49^ As reported, FABP4 downregulation possibly resulted in increasing free FA availability, therefore, mitochondrial functions such as lipolysis and ATP production were inversely enhanced through rescue mechanisms such as the increase of the rate of FA oxidation (FAO), tricarboxylic acid (TCA) cycling, as well as oxidative phosphorylation. In our study, the level and function of ATP were also investigated. Interestingly, we further found that activation of the NF‐κB/RelA‐IL1α axis closely depends on FABP4 knockdown‐induced ATP production. Mitochondria ATP is responsible for the cell's energy requirements. Moreover, it also actively regulates innate immune responses against infectious and sterile insults.^50^ Excluding the rescue mechanisms, we wondered whether ATP levels induced by FABP4 resulted from the interaction between FABP4 and ATPB. ATPB is predicted to contain several ubiquitinate sites, including proteasomal degradation (http://cplm.biocuckoo.org). Our study verified that FABP4 is responsible for ATPB stability, its overexpression increased the binding activity with the *N*‐terminal fragment of ATPB and impeded the interaction with USP30, leading to subsequent ubiquitination. Collectively, these findings further delineated the detailed mechanism underlying FABP4‐mediated ATPB activity and NF‐κB translocation. Although intratumor inflammation has provided a validated prognostic signature for children with metastatic NBL‐NA,^10^ the molecular mechanisms underlying NB were largely unknown. More effective therapeutic strategies targeting immune cells could be used to reduce therapy‐related toxicities of high‐risk NB.

In summary, our study suggests expression of FABP4 in macrophages is an independent prognostic factor and predicts poor outcomes in NB patients, complementary to MYCN status and clinical stage. Our results also indicate that FABP4 in macrophages increases malignant phenotypes of NB cells in vitro and tumor growth in vivo, whereas knockdown of FABP4 in macrophages suppresses NB cell invasive properties. Mechanistically, FABP4 promotes ATPB ubiquitination, decreases ATP levels, and deactivates NF‐κB/RelA‐IL1α pathway, to sustain macrophages in an anti‐inflammatory phenotype in NB. Thus, FABP4 may serve as a new functional marker for protumor TAM in NB, and targeting FABP4 in TAM may represent a novel strategy for NB immunotherapy.

## 4 METHODS

### 4.1 Cell isolation and cell culture

Human HUVEC, THP1, and NB cell lines SK‐N‐BE and SK‐N‐SH were obtained from the American Type Culture Collection (ATCC, Manassas, VA, USA) and maintained in RPMI‐1640 containing 10% fetal bovine serum (FBS) and 1% penicillin/streptomycin at 37°C under 5% CO_2_. All the cells were authenticated via short‐tandem repeat profiling and tested free of mycoplasma contamination.

PBMCs were provided and isolated by Ficoll‐Paque Plus reagent (#45‐001‐749; GE Healthcare, Pittsburgh, PA, USA). Cell viability was determined by trypan blue staining. CD14^+^ monocytes were further isolated by anti‐CD14 microbeads (#130‐050‐201; Miltenyi Biotec, Bergisch Gladbach, Germany) and cultured in RPMI‐1640 medium containing 10% FBS. The monocytes were further stimulated with 20 ng/mL M‐CSF (#30025; PeproTech, Rocky Hill, USA) for 7–10 days to obtain mature macrophages (PBMC‐DMs). THP1 cells were plated and stimulated by phorbol 12‐myristate 13‐acetate (PMA, #P8139; Sigma‐Aldrich, St. Louis, USA) for 4 h or overnight to obtain THP1‐DMs.

CD3^+^ T cells were isolated from PBMCs by a Pan T Cell Isolation Kit (#130‐096‐535; MiltenyiBiotec, Bergisch Gladbach, Germany), and further classified as CD4^+^ or CD8^+^ T cells by using CD4 or CD8 microbeads (#130‐045‐101 or #130‐045‐201; MiltenyiBiotec). All the T cells were cultured in T‐cell expansion medium (ImmunoCult‐XF T Cell Exp Medium; StemCell, Vancouver, Canada).

### 4.2 NB tissue samples

NB tissue samples were obtained from Guangzhou Women and Children's Medical Center. Ethical approval was obtained from the Institutional Ethics Committee and written consent was obtained from patients’ guardians before collection of samples. NB samples were categorized into two groups—early‐stage (stages I and II, *n* = 10) and advanced‐stage (stages III and IV, *n* = 10) groups—based on the clinicopathological information. We adopted another fresh early‐stage (*n* = 5) and advanced‐stage (*n* = 5) NB samples for TAMs and classic monocytes analysis in tissues and PBMCs. This study was conducted in accordance with the ethical standards of the World Medical Association Declaration of Helsinki.

### 4.3 IHC and immunofluorescence

NB tissue samples and mice tumor samples were fixed and embedded for IHC staining as previously described.^51^ After blockade, the indicated sections were separately incubated with rabbit anti‐FABP4 antibody (#ab13979; Abcam, Cambridge, MA, USA), mouse anti‐human CD68 antibody (#ab201340; Abcam), mouse anti‐CD8 antibody (#66868‐1‐Ig; ProteinTech, Chicago, IL, USA), rabbit anti‐CD4 antibody (#25229; Cell Signaling Technology, Beverly, USA), rabbit anti‐Ki67 antibody (Cat#12202; Cell Signaling Technology), rabbit anti‐Twist antibody (#25465‐1‐AP; ProteinTech), rabbit anti‐ATPB antibody (#ab14730; Abcam), and mouse anti‐IL1α antibody (#MAB200‐SP; R&D, MN, USA) at 4°C overnight, followed by hybridized with biotinylated goat anti‐rabbit/mouse immunoglobulin (GK500705; Dako, Glostrup, Denmark) at 37°C for 30 min and visualized with diaminobenzidine (DAB) (GK500705; Dako). Five representative fields of each section were captured and analyzed. The average positive ratio was defined by the symbol and color. The positive ratios of CD4 and CD8 were defined by the symbol and color in nucleated cells. The slides were scored independently by two experienced pathologists.

Immunofluorescence was conducted as described previously.^52^ The tissue sections or fixed cells were incubated with anti‐FABP4 antibody, anti‐human CD68 antibody, and rabbit anti‐ATPB antibody, independently, followed by incubating with anti‐mouse or rabbit Alexa Fluor secondary antibodies (#4410 or #4412; Cell Signaling Technology). The nuclei were visualized with DAPI staining. All images were acquired by confocal microscopy (Leica SP8; Lecia Microsystems, Germany) equipped with a 40× objective.

### 4.4 Reagents and preparation of CM

MG‐132 (HY‐13259), Bay 11–7082 (HY‐13453), Oligomycin A (HY‐16589), and protein A/G magnetic beads (HY‐K0202) were purchased from MedChemExpress (New Jersey, USA). Cycloheximide (CHX) was purchased from Abmole (TX, USA). Human CXCL8 (#200‐08 M) and IL‐1α (#200‐01A) were purchased from PeproTech. Mouse anti‐CXCL8 antibody (#MA5‐23697) was purchased from Thermo Fisher Scientific (Waltham, MA, USA).

For preparing macrophages‐derived CMs, THP1‐DMs were seeded in culture medium for 24 h, thereafter exposing to various treatments for 48 h, the supernatants were collected and stored.

### 4.5 Cell transfection

Human FLAG‐tagged cDNAs of ATPB, including FL (1‐529aa), *N*‐Terminal (1‐265aa), *C*‐Terminal (266‐529aa), and full‐length (FL) FABP4 were all synthesized by OBiO Technology Inc. (Shanghai, China) and subcloned into pcDNA3.1 vector. The wide‐type (wt) and deletion (dt) cDNAs of IL1α promoter region (944‐953: GGGGCTTCACTATGTT) were constructed in pGL4.10 vector. The shRNA sequences targeting human FABP4 (#1: GCATGGCCAAACCTAACAT; #2: GGAAAGTCAAGAGCACCAT; #3: CCTGGTACATGTGCAGAAA) were assembled into the vector pDKD‐CMV‐Puro‐U6‐shRNA and validated by RT‐PCR. Cell transfection was performed using Lipofectamine 3000 Transfection Reagent (Invitrogen, Carlsbad, CA, USA). As for THP1‐DMs, NATE (#lyec‐nate; Invivogen, MA, USA) was added to enhance transfection efficacy. The scrambled shRNA sequence (SC: TTCTCCGAACGTGTCACGT) was used as a control for FABP4 knockdown, while the empty vector was constructed as a control for FABP4 overexpression. Human FABP4 cDNA and selected shRNA (Sh2‐FABP4) plasmids were also packaged into adenoviruses (pADV‐U6‐shRNA‐CMV‐MCS) by OBiO Technology Inc. for PBMC‐DMs transfection.

### 4.6 Flow cytometry analysis

Cell cycle analysis was performed with a cell cycle detection kit (#KGA511; KeyGen, Nanjing, China) according to the manufacturer's instructions. The treated cells were fixed with 75% ethanol and then stained with PI for flow cytometry analysis.

To detect the phenotype of PBMC‐DMs, cells were incubated with indicated CMs for 48 h after differentiation. The phenotype of the macrophages was analyzed by flow cytometry, independently, followed by staining with APC anti‐human CD209 (#330107), FITC anti‐human CD80 (#305205), APC anti‐human CCR7 (#353213), PE anti‐human CD11b (#301305), FITC anti‐human CD36 (336203), Alex Fluor^@^700 anti‐human CD16 (#302026), PerCP anti‐human CD14 (#325632), PE/Cyanine 7 anti‐human CD45 (#368532), Brilliant Violet 421 anti‐human HLA‐DR (#307635), and APC anti‐human Lineage cocktail (CD3, CD19, CD20, CD56) (#363601), which were all purchased from BioLegend (San Diego, CA, USA).

### 4.7 Cell proliferation

Cell proliferation was measured by CCK8 assay (Dojindo, Japan) according to the manufacturer's instructions. The SK‐N‐BE and SK‐N‐SH cells were plated in 96‐well plates for 24 h and treated as indicated. The cell viability index was obtained by calculating absorbance at 492 nm.

### 4.8 Cell migration and wound healing assay

For cell migration assay, cells (1 × 10^5^ per well) were seeded in the upper inserts for 24 h and replaced with serum‐free RPMI‐1640 in 24‐well plates. The inserts were then exposed to the lower chambers within RPMI‐1640 containing 2% FBS and 20% THP1‐DMs treated CMs. RPMI‐1640 containing 2% FBS within the bottom was used as the control. After 12 h, the cells that migrated onto the lower surface of the membrane were counted and analyzed using Image J software.

As for the wound healing assay, SK‐N‐BE and SK‐N‐SH cells were seeded in six‐well plates and incubated to reach 95% confluence. The monolayer was scratched using a tip and washed with a serum‐free medium to remove detached cells. After that, cells were exposed to various mediums containing 20% PBMC‐DMs treated CMs and incubated for 24, 48, and 72 h, respectively. Cell motility in each group was assessed by visualizing the speed of wound closure at intervals.

### 4.9 Detection of ATP content

PBMC‐DMs and THP1‐DMs treated as indicated were lysed. The ATP levels were detected using an enhanced ATP assay kit (#S0027; Beyotime, Nanjing, China), according to the protocol provided by the manufacturer.

### 4.10 Chemotaxis assay of T cells

Purified CD4^+^ or CD8^+^ T cells (1 × 10^6^ per well) were seeded in the upper chambers of a 24‐well transwell plate (Corning, NY, USA). The THP1‐DMs treated CMs were added to the lower chambers (inserts with 0.4 mm pore size) for 12 h. The T‐cell expansion medium within the bottom was used as the control. The chemotaxis of CD4^+^ or CD8^+^ T cells was analyzed by counting the number of migrated cells.

### 4.11 Western blot

Western blot was performed according to standard procedure. The treated cells were lysed with ice‐cold RIPA buffer containing both protease and phosphatase inhibitors. The cytoplasmic and nuclear extracts from cells were separated using nuclear and cytoplasmic Extraction kits (#78833; Thermo Fisher Scientific). Protein concentrations were quantified using a BCA protein assay kit (Thermo Fisher Scientific). Primary antibodies including anti‐β‐actin (#4790), anti‐FABP4, anti‐CD68, anti‐PCNA (#13110), anti‐p21 (#2974), anti‐Cyclin B1 (#12231), anti‐NF‐κB/p65 (#6956), anti‐Histone H3 (#4499), anti‐FLAG tag (#8146), anti‐ubiquitin antibodies (#3933), and FAK antibody sample kit (#9330) were all purchased from cell signaling technology (Beverly, USA). Anti‐March5 antibody was purchased from ProteinTech (#12213‐1‐AP). Anti‐human USP30 antibody was purchased from Abcam (#ab235299). All bands were visualized by enhanced chemiluminescence and quantified by Image J analysis software.

### 4.12 RNA extraction and RT‐PCR

Total RNA was obtained using an RNA extraction kit (#RN001; ESscience, Shanghai, China) and converted to cDNA according to the manufacturer's instructions (#RR036A‐1; TAKARA, Japan). The relative expressions of identified genes were performed and measured by quantitative real‐time PCR assays (ABI Q6 System, Applied Biosystems). All the genes were normalized to GAPDH, and the fold change was calculated as 2^–ΔΔCT^ comparative thresholds. Specific primer sequences are shown in Table S1.

### 4.13 Enzyme‐linked immunosorbent assay

PBMC‐DMs and THP1‐DMs were seeded in culture medium for 24 h, thereafter exposing to various treatments for 48 h. The supernatants were collected for measurement of the levels of IL1α and secreted FABP4 by using human IL1α ELISA Kit (#SEA071Hu; Cloud‐Clone Corp, Hubei, China) and FABP4 ELISA Kit (#SEB693Hu; Cloud‐Clone Corp), respectively.

### 4.14 ChIP, IP, Co‐IP, and luciferase reporter assays

ChIP assays were performed with a Simple CHIP kit (#66816; Cell Signaling Technology) according to the protocol provided by the manufacturer. Anti‐NF‐κB/p65 was used in the ChIP assays. The products were further labeled with Simple CHIP qPCR Master Mix (#88989; Cell Signaling Technology) for quantitative real‐time PCR assays to identify the DNA region immunoprecipitated with NF‐κB.

Anti‐FABP4 and ATPB were used for IP and Co‐IP assays, and the immune precipitants were detected by immunoblotting. Anti‐rabbit and mouse IgG (#3900 and #3420; Cell Signaling Technology) were used as negative controls.

For luciferase reporter assays (#N1620; Promega, WI, USA), firefly and Renilla Luciferase activities were examined by the Dual‐Luciferase Reporter Assay System, and the firefly activity was used to normalize Renilla activity.

### 4.15 In vivo study

SK‐N‐SH‐derived orthotopic and metastatic NB models were generated by the left adrenal gland or tail‐vein injection with SK‐N‐SH cells expressing inducible luciferase reporter, respectively. The procedure was shown in Figure S4A. After irradiation (0.5 Gy), the mice were administrated clodronate liposomes (CL2MDP, Clodronate Liposomes.org, LB, The Netherlands) intraperitoneally to deplete instinct macrophages, followed by transplantation of 5 × 10^6^ human PBMCs. PBS liposomes were administrated as a negative control. The efficiency of macrophage depletion was verified and described in the result section. All the mice were randomly assigned into five groups (*n* = 10 per group): no PBMC‐DMs, mice transplanted with PBS liposomes, control PBMC‐DMs, shFABP4‐treated PBMC‐DMs, and FABP4‐treated PBMC‐DMs, respectively. The tumor size was monitored by luciferase imaging per week using the In‐Vivo BRUKER FX PRO. Luciferase intensity was also captured and measured. As for the metastatic model, metastases nodes were also counted and analyzed. Three weeks after transplantation, the mice were sacrificed, and tumor tissues were collected for further pathological analysis. All the animal experiments were approved by the Institutional Animal Care and Use Committee of Sun Yat‐sen University Cancer Center.

### 4.16 Statistical analysis

All the Western blots, immunofluorescence, and IHC images are representative results from at least two independent biological replicates. Correlations between IL1α level and FABP4 expression were analyzed with Pearson's correlation analysis. All bar graphs represent the mean ± SD. Statistical calculations were derived from at least three independent experiments and analyzed by Student's *t*‐test (unpaired, two‐tailed) or one‐way ANOVA using GraphPad Prism 8.0.1 software (GraphPad, La Jolla, CA, USA). The *p‐*value < 0.05 indicated statistical significance: *p < 0.05, **p < 0.01, ***p < 0.001.

## AUTHOR CONTRIBUTIONS

Lei Miao, Jing He, and Huimin Xia designed the study. Lei Miao, Zhenjian Zhuo, and Jiabin Liu completed in vitro experiments. Jue Tang and Hai‐Yun Wang collected NB samples and clinical data. Xiaomei Huang collected human whole blood for PBMC isolation. Lei Miao and Zhenjian Zhuo analyzed the bioinformatics data. Lei Miao and Jing He collected and statistically analyzed the data. Lei Miao, Zhenjian Zhuo, and Jue Tang wrote the manuscript and contributed equally to the study. All authors reviewed the manuscript and approved the final version.

## CONFLICT OF INTEREST

The authors declare that there is no conflict of interest.

## Supporting information

TABLE S1 These primers used in RT‐qPCR

## References

[1] Brodeur GM . Neuroblastoma: biological insights into a clinical enigma. Nat Rev Cancer. 2003;3:203‐216. 12612655 10.1038/nrc1014

[2] DuBois SG , Kalika Y , Lukens JN , et al. Metastatic sites in stage IV and IVS neuroblastoma correlate with age, tumor biology, and survival. J Pediatr Hematol Oncol. 1999;21:181‐189. 10363850 10.1097/00043426-199905000-00005

[3] Maris JM . Recent advances in neuroblastoma. N Engl J Med. 2010;362:2202‐2211. 20558371 10.1056/NEJMra0804577 PMC3306838

[4] Yang RK , Sondel PM . Anti‐GD2 strategy in the treatment of neuroblastoma. Drugs Future. 2010;35:665. 21037966 10.1358/dof.2010.035.08.1513490 PMC2964668

[5] Condeelis JS , Pollard JW . Macrophages: obligate partners for tumor cell migration, invasion, and metastasis. Cell. 2006;124:263‐266. 16439202 10.1016/j.cell.2006.01.007

[6] Stout RD , Jiang C , Matta B , Tietzel I , Watkins SK , Suttles J . Macrophages sequentially change their functional phenotype in response to changes in microenvironmental influences. J Immunol. 2005;175:342‐349. 15972667 10.4049/jimmunol.175.1.342

[7] Tian K , Wang X , Wu Y , et al. Relationship of tumour‐associated macrophages with poor prognosis in Wilms' tumour. J Pediatr Urol. 2020;16:376.e1‐376.e8. 32299765 10.1016/j.jpurol.2020.03.016

[8] Sawa‐Wejksza K , Kandefer‐Szerszeń M . Tumor‐associated macrophages as target for antitumor therapy. Arch Immunol Ther Exp. 2018;66:97‐111. 10.1007/s00005-017-0480-8 PMC5851686 28660349

[9] Dammeijer F , Lievense LA , Kaijen‐Lambers ME , et al. Depletion of tumor‐associated macrophages with a CSF‐1R kinase inhibitor enhances antitumor immunity and survival induced by DC immunotherapy. Cancer Immunol Res. 2017;5:535‐546. 28536100 10.1158/2326-6066.CIR-16-0309

[10] Asgharzadeh S , Salo JA , Ji L , et al. Clinical significance of tumor‐associated inflammatory cells in metastatic neuroblastoma. J Clin Oncol. 2012;30:3525‐3532. 22927533 10.1200/JCO.2011.40.9169 PMC3675667

[11] Larsson K , Kock A , Idborg H , et al. COX/mPGES‐1/PGE2 pathway depicts an inflammatory‐dependent high‐risk neuroblastoma subset. Proc Natl Acad Sci. 2015;112:8070‐8075. 26080408 10.1073/pnas.1424355112 PMC4491767

[12] Abraham D , Zins K , Sioud M , et al. Stromal cell‐derived CSF‐1 blockade prolongs xenograft survival of CSF‐1‐negative neuroblastoma. Int J Cancer. 2010;126:1339‐1352. 19711348 10.1002/ijc.24859 PMC3222589

[13] Liu Q , Luo Q , Halim A , Song G . Targeting lipid metabolism of cancer cells: a promising therapeutic strategy for cancer. Cancer Lett. 2017;401:39‐45. 28527945 10.1016/j.canlet.2017.05.002

[14] Furuhashi M , Hotamisligil GS . Fatty acid‐binding proteins: role in metabolic diseases and potential as drug targets. Nat Rev Drug Discovery. 2008;7:489‐503. 18511927 10.1038/nrd2589 PMC2821027

[15] Levi L , Wang Z , Doud MK , Hazen SL , Noy N . Saturated fatty acids regulate retinoic acid signalling and suppress tumorigenesis by targeting fatty acid‐binding protein 5. Nat Commun. 2015;6:8794‐8794. 26592976 10.1038/ncomms9794 PMC4662070

[16] Uehara H , Kobayashi T , Matsumoto M , Watanabe S , Yoneda A , Yoshimi B . Adipose tissue: critical contributor to the development of prostate cancer. J Med Invest. 2018;65:9‐17. 29593201 10.2152/jmi.65.9

[17] Hao J , Zhang Y , Yan X , et al. Circulating adipose fatty acid binding protein is a new link underlying obesity‐associated breast/mammary tumor development. Cell Metab. 2018;28:689‐705.e5. 30100196 10.1016/j.cmet.2018.07.006 PMC6221972

[18] Thompson KJ , Austin RG , Nazari SS , Gersin KS , Iannitti DA , McKillop IH . Altered fatty acid‐binding protein 4 (FABP 4) expression and function in human and animal models of hepatocellular carcinoma. Liver Int. 2018;38:1074‐1083. 29171144 10.1111/liv.13639

[19] Elmasri H , Ghelfi E , Yu C‐W , et al. Endothelial cell‐fatty acid binding protein 4 promotes angiogenesis: role of stem cell factor/c‐kit pathway. Angiogenesis. 2012;15:457‐468. 22562362 10.1007/s10456-012-9274-0 PMC3590918

[20] Nieman KM , Kenny HA , Penicka CV , et al. Adipocytes promote ovarian cancer metastasis and provide energy for rapid tumor growth. Nat Med. 2011;17:1498. 22037646 10.1038/nm.2492 PMC4157349

[21] Makowski L , Brittingham KC , Reynolds JM , Suttles J , Hotamisligil GS . The fatty acid‐binding protein, aP2, coordinates macrophage cholesterol trafficking and inflammatory activity macrophage expression of aP2 impacts peroxisome proliferator‐activated receptor γ and IκB kinase activities. J Biol Chem. 2005;280:12888‐12895. 15684432 10.1074/jbc.M413788200 PMC3493120

[22] Liang X , Gupta K , Quintero JR , et al. Macrophage FABP4 is required for neutrophil recruitment and bacterial clearance in Pseudomonas aeruginosa pneumonia. FASEB J. 2019;33:3562‐3574. 30462529 10.1096/fj.201802002R PMC6988858

[23] Harjes U , Bridges E , Gharpure K , et al. Antiangiogenic and tumour inhibitory effects of downregulating tumour endothelial FABP4. Oncogene. 2017;36:912‐921. 27568980 10.1038/onc.2016.256 PMC5318662

[24] Asgharzadeh S , Pique‐Regi R , Sposto R , et al. Prognostic significance of gene expression profiles of metastatic neuroblastomas lacking MYCN gene amplification. J Natl Cancer Inst. 2006;98:1193‐1203. 16954472 10.1093/jnci/djj330

[25] Mita T , Furuhashi M , Hiramitsu S , et al. FABP4 is secreted from adipocytes by adenyl cyclase‐PKA‐ and guanylyl cyclase‐PKG‐dependent lipolytic mechanisms. Obesity (Silver Spring). 2015;23:359‐367. 25521833 10.1002/oby.20954

[26] Hashimoto O , Yoshida M , Koma Y , et al. Collaboration of cancer‐associated fibroblasts and tumour‐associated macrophages for neuroblastoma development. J Pathol. 2016;240:211‐223. 27425378 10.1002/path.4769 PMC5095779

[27] Wang Y , Guo W , Xu H , et al. An extensive study of the mechanism of prostate cancer metastasis. Neoplasma. 2018;65:253‐261. 29534587 10.4149/neo_2018_161217N648

[28] Kuehnemuth B , Piseddu I , Wiedemann GM , et al. CCL1 is a major regulatory T cell attracting factor in human breast cancer. BMC Cancer. 2018;18:1278. 30572845 10.1186/s12885-018-5117-8 PMC6302432

[29] Kurose K , Ohue Y , Sato E , et al. Increase in activated Treg in TIL in lung cancer and in vitro depletion of Treg by ADCC using an antihuman CCR4 mAb (KM2760). J Thorac Oncol. 2015;10:74‐83. 25325779 10.1097/JTO.0000000000000364

[30] Bouchet M , Lainé A , Boyault C , et al. ERRα expression in bone metastases leads to an exacerbated antitumor immune response. Cancer Res. 2020;80:2914‐2926. 32366476 10.1158/0008-5472.CAN-19-3584

[31] Di Paolo NC , Shayakhmetov DM . Interleukin 1alpha and the inflammatory process. Nat Immunol. 2016;17:906‐913. 27434011 10.1038/ni.3503 PMC5152572

[32] Hess C , Means TK , Autissier P , et al. IL‐8 responsiveness defines a subset of CD8 T cells poised to kill. Blood. 2004;104:3463‐3471. 15292066 10.1182/blood-2004-03-1067

[33] Hu H , Miao YR , Jia LH , Yu QY , Zhang Q , Guo AY . AnimalTFDB 3.0: a comprehensive resource for annotation and prediction of animal transcription factors. Nucleic Acids Res. 2019;47:D33‐D38. 30204897 10.1093/nar/gky822 PMC6323978

[34] Fornes O , Castro‐Mondragon JA , Khan A , et al. JASPAR 2020: update of the open‐access database of transcription factor binding profiles. Nucleic Acids Res. 2020;48:D87‐D92. 31701148 10.1093/nar/gkz1001 PMC7145627

[35] Chelko SP , Asimaki A , Lowenthal J , et al. Therapeutic modulation of the immune response in arrhythmogenic cardiomyopathy. Circulation. 2019;140:1491‐1505. 31533459 10.1161/CIRCULATIONAHA.119.040676 PMC6817418

[36] Li W , Li Y , Li G , et al. Ectopic expression of the ATP synthase β subunit on the membrane of PC‐3M cells supports its potential role in prostate cancer metastasis. Int J Oncol. 2017;50:1312‐1320. 28259978 10.3892/ijo.2017.3878

[37] Gale M , Li Y , Cao J , et al. Acquired resistance to HER2‐targeted therapies creates vulnerability to ATP synthase inhibition. Cancer Res. 2020;80:524‐535. 31690671 10.1158/0008-5472.CAN-18-3985 PMC7002225

[38] Phu L , Rose CM , Tea JS , et al. Dynamic regulation of mitochondrial import by the ubiquitin system. Mol Cell. 2020;77:1107‐1123.e10. 32142684 10.1016/j.molcel.2020.02.012

[39] Relation T , Yi T , Guess AJ , et al. Intratumoral delivery of interferonγ‐secreting mesenchymal stromal cells repolarizes tumor‐associated macrophages and suppresses neuroblastoma proliferation in vivo. Stem Cells. 2018;36:915‐924. 29430789 10.1002/stem.2801

[40] Joshi S . Targeting the tumor microenvironment in neuroblastoma: recent advances and future directions. Cancers. 2020;12:2057. 10.3390/cancers12082057 PMC7465822 32722460

[41] Iwamoto H , Abe M , Yang Y , et al. Cancer lipid metabolism confers antiangiogenic drug resistance. Cell Metab. 2018;28:104‐117.e5. 29861385 10.1016/j.cmet.2018.05.005

[42] Erbay E , Babaev VR , Mayers JR , et al. Reducing endoplasmic reticulum stress through a macrophage lipid chaperone alleviates atherosclerosis. Nat Med. 2009;15:1383. 19966778 10.1038/nm.2067 PMC2790330

[43] Furuhashi M , Saitoh S , Shimamoto K , Miura T . Fatty acid‐binding protein 4 (FABP4): pathophysiological insights and potent clinical biomarker of metabolic and cardiovascular diseases. Clin Med Insights Cardiol. 2014;8:CMC. S17067. 10.4137/CMC.S17067 PMC4315049 25674026

[44] Ishimura S , Furuhashi M , Watanabe Y , et al. Circulating levels of fatty acid‐binding protein family and metabolic phenotype in the general population. PLoS One. 2013;8. 10.1371/journal.pone.0081318 PMC3835575 24278421

[45] Demaria O , De Gassart A , Coso S , et al. STING activation of tumor endothelial cells initiates spontaneous and therapeutic antitumor immunity. PNAS. 2015;112:15408‐15413. 26607445 10.1073/pnas.1512832112 PMC4687570

[46] Voronov E , Dinarello CA , Apte RN . Interleukin‐1α as an intracellular alarmin in cancer biology. Semin Immunol. 2018;38:3‐14. 30554608 10.1016/j.smim.2018.10.006

[47] Voronov E , Dotan S , Krelin Y , et al. Unique versus redundant functions of IL‐1α and IL‐1β in the tumor microenvironment. Front Immunol. 2013;4:177. 23847618 10.3389/fimmu.2013.00177 PMC3703603

[48] Gillilan RE , Ayers SD , Noy N . Structural basis for activation of fatty acid‐binding protein 4. J Mol Biol. 2007;372:1246‐1260. 17761196 10.1016/j.jmb.2007.07.040 PMC2032018

[49] Anjum K , Bi H , Chai W , Lian XY , Zhang Z . Antiglioma pseurotin A from marine bacillus sp. FS8D regulating tumour metabolic enzymes. Nat Prod Res. 2018;32:1353‐1356. 28641457 10.1080/14786419.2017.1343329

[50] Banoth B , Cassel SL . Mitochondria in innate immune signaling. Transl Res. 2018;202:52‐68. 30165038 10.1016/j.trsl.2018.07.014 PMC6218307

[51] Miao L , Wei XL , Zhao Q , et al. P476S mutation of RBPJL inhibits the efficacy of anti‐PD‐1 therapy in oesophageal squamous cell carcinoma by blunting T‐cell responses. Clin Transl Immunol. 2020;9:e1172. 10.1002/cti2.1172 PMC7507108 32994998

[52] Miao L , Qi J , Zhao Q , et al. Targeting the STING pathway in tumor‐associated macrophages regulates innate immune sensing of gastric cancer cells. Theranostics. 2020;10:498‐515. 31903134 10.7150/thno.37745 PMC6929973

