# Supplementary material for: FABP4 deactivates NF‐κB‐IL1α pathway by ubiquitinating ATPB in tumor‐associated macrophages and promotes neuroblastoma progression
Source: Clin Transl Med. 2021 May 1;11(4):e395. doi: 10.1002/ctm2.395 (PMC8087928; doi:10.1002/ctm2.395)
Supplement: Supplementary file 1 — TABLE S1 These primers used in RT‐qPCR [file CTM2-11-e395-s001.docx]

| **Table S1** These primers used in RT-qPCR | | |
| --- | --- | --- |
| **Name** | **Forward primer** | **Reverse primer** |
| *Homo* β actin | CTACCTCATGAAGATCCTCACCGA | TTCTCCTTAATGTCACGCACGATT |
| *Homo* CRABP1 | GCAGCAGCGAGAATTTCGAC | CGTGGTGGATGTCTTGATGTAGA |
| *Homo* PMP2 | GACGATTACATGAAAGCTCTGGG | TGCACTTGATTCAGTGATCCTCT |
| *Homo* RARRES2 | AGAAACCCGAGTGCAAAGTCA | AGAACTTGGGTCTCTATGGGG |
| *Homo* FABP4 | ACTGGGCCAGGAATTTGACG | CTCGTGGAAGTGACGCCTT |
| *Homo* ADIPOQ | TGCTGGGAGCTGTTCTACTG | TACTCCGGTTTCACCGATGTC |
| *Homo* TUSC5 | CCACCTCCTATGCCCAAGAC | GCTGCTTCGAGACATGATGGAA |
| *Homo* PLIN1 | TGTGCAATGCCTATGAGAAGG | AGGGCGGGGATCTTTTCCT |
| *Homo* LEP | TGCCTTCCAGAAACGTGATCC | CTCTGTGGAGTAGCCTGAAGC |
| *Homo* LIPE | TCAGTGTCTAGGTCAGACTGG | AGGCTTCTGTTGGGTATTGGA |
| *Homo* CEL | CCTGTACCTCAACATTTGGGTG | GATGACGTTTCCGCGTGTG |
| *Homo* APOE | GTTGCTGGTCACATTCCTGG | GCAGGTAATCCCAAAAGCGAC |
| *Homo* CIDEC | AAGTCCCTTAGCCTTCTCTACC | CCTTCCTCACGCTTCGATCC |
| *Homo* IL1A | TGGTAGTAGCAACCAACGGGA | ACTTTGATTGAGGGCGTCATTC |
| *Homo* CCL2 | CAGCCAGATGCAATCAATGCC | TGGAATCCTGAACCCACTTCT |
| *Homo* CXCL3 | CGCCCAAACCGAAGTCATAG | GCTCCCCTTGTTCAGTATCTTTT |
| *Homo* IL6 | ACTCACCTCTTCAGAACGAATTG | CCATCTTTGGAAGGTTCAGGTTG |
| *Homo* CXCL8 | ACTGAGAGTGATTGAGAGTGGAC | AACCCTCTGCACCCAGTTTTC |
| *Homo* IL1B | AGCTACGAATCTCCGACCAC | CGTTATCCCATGTGTCGAAGAA |
| *Homo* IL23A | CTCAGGGACAACAGTCAGTTC | ACAGGGCTATCAGGGAGCA |
| *Homo* CXCL1 | TCACAGTGTGTGGTCAACAT | AGCCCCTTTGTTCTAAGCCA |
| *Homo* IL1A-BR (944-953) | TCACTTGTGCAGTGTTGACAG | ACAAGGCTCAGTACATGCTCA |
| *Homo* ATPB | CCTGTCAGGGACTATGCGG | TCCTTACTGTGCTCTCACCCA |
| *Homo* CPT1A | ATCAATCGGACTCTGGAAACGG | TCAGGGAGTAGCGCATGGT |
